# High-Resolution Magic Angle Spinning NMR of KcsA in Liposomes: The Highly Mobile C-Terminus

**DOI:** 10.3390/biom12081122

**Published:** 2022-08-15

**Authors:** Gary S. Howarth, Ann E. McDermott

**Affiliations:** Department of Chemistry, Columbia University, New York, NY 10031, USA

**Keywords:** solid-state nuclear magnetic resonance (SS NMR), high-resolution nuclear magnetic resonance (HR-MAS NMR), KcsA, liposomes, lipid chemical shifts, lipid hydrolysis, membrane protein structure

## Abstract

The structure of the transmembrane domain of the pH-activated bacterial potassium channel KcsA has been extensively characterized, yet little information is available on the structure of its cytosolic, functionally critical N- and C-termini. This study presents high-resolution magic angle spinning (HR-MAS) and fractional deuteration as tools to study these poorly resolved regions for proteoliposome-embedded KcsA. Using ^1^H-detected HR-MAS NMR, we show that the C-terminus transitions from a rigid structure to a more dynamic structure as the solution is rendered acidic. We make previously unreported assignments of residues in the C-terminus of lipid-embedded channels. These data agree with functional models of the C-terminus-stabilizing KcsA tetramers at a neutral pH with decreased stabilization effects at acidic pH. We present evidence that a C-terminal truncation mutation has a destabilizing effect on the KcsA selectivity filter. Finally, we show evidence of hydrolysis of lipids in proteoliposome samples during typical experimental timeframes.

## 1. Introduction

KcsA, a pH-activated, inward-rectifying potassium channel from the Gram-positive soil bacterium *Streptomyces lividans*, has played a unique role in the structural biology of ion channels, being the first to be characterized in detail. The continuing progress on structural, biochemical, electrophysiological, and biophysical characterization of KcsA makes it a uniquely rich model system for transmembrane allosteric coupling [2,3], ion channel activation and inactivation mechanism [4,5], lipid–protein interactions [6,7], and virtually any other question concerning ion channels and membrane proteins. However, we do not yet have a full-length, atomic-resolution structure of KcsA in a lipid membrane.

In situ, the KcsA channel is closed at neutral pH, briefly activates to let K^+^ ions flow into the cell when the cytosol is rendered acidic, and then enters an inactivation period. Functional channels are composed of four identical subunits of 160 amino acids, each with two transmembrane-spanning domains. While the transmembrane segments are relatively well-characterized, the most mobile portions of KcsA, its extracellular termini, present a unique set of challenges to resolve while in the membrane, despite their important roles.

Several efforts [5,8,9,10,11], most notably by Chill et al. [12], have provided nearly complete assignments for KcsA in detergent micelles. Solution studies have provided remarkable information about the dynamics [13], secondary structure [12], and the major [14] and minor pH sensors [15]. Solution studies of KcsA often involve truncation mutants and high temperatures (e.g., >45 °C).

Logically, channels can only be considered functional in a lipid bilayer. KcsA activation is lipid-dependent. Therefore, details of the lipid environment are likely to be important to structural elements relevant to function. KcsA has been shown to have an open-probability dependence on the lipid headgroup with a requirement of anionic lipids in the inner leaflet [6]. The stability of the KcsA tetramer is also lipid-dependent with anionic headgroups (e.g., phosphatidic acid (PA), phosphoglycerol (PG), and phosphoserine (PS)) providing greater stability, especially at a low pH compared to lipids with net-neutral or net-positive headgroups [16]. Crystal structures [17,18,19,20], biochemical experiments [21], and NMR data [22] have shown that KcsA routinely co-purifies with a diacyl lipid with a PG headgroup. Protein–lipid affinity experiments have shown that KcsA has a high affinity for the net-negatively charged lipids (PA, PE, and PG), with PG affinity being the highest of all [23].

**Figure 1:**
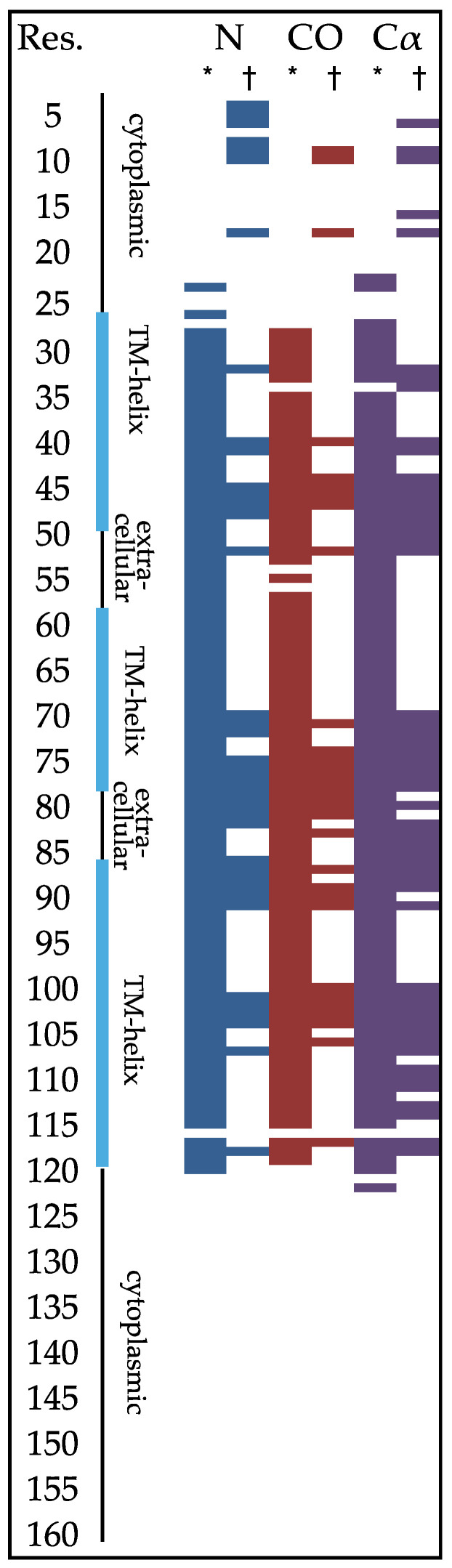
Solid-state NMR backbone assignments of KcsA in liposomes as described by the McDermott Group (*) [3,26,33,34] or Baldus Group (†) [28,35,36]. Structural regions are indicated to the right of residue numbers.

High-resolution cross-polarization magic angle spinning solid-state NMR (CP-MAS) has emerged as one of the few techniques that can investigate membrane channels embedded in a lipid bilayer. There have been many successful efforts to use CP-MAS to make residue assignments to resonances of KcsA in proteoliposomes [24,25,26,27,28]. The protein residues that are detected and assigned are notably incomplete, emphasizing primarily the transmembrane helices (Figure 1). Yet, unobserved regions of KcsA are functionally critical.

### The KcsA C-Terminus

The KcsA C-terminus is an example of a critical region that remains unresolved when channels are in a lipid membrane. The C-terminus of KcsA has been proposed to have a high degree of α-helix character and to project perpendicular to the membrane surface [1,12,29,30]. Truncations of and mutations within the C-terminus lead to reduced tetramer stability [31,32] and affect gating and pH dependence [15,29,32].

Where biochemical experiments have indicated the functional importance of KcsA’s C-terminal domain [5,8,9,10,11], structural studies provide insight into the potential mechanism by which the C-terminus regulates KcsA function. Recombinant KcsA expressed as only as its C-terminal domain is soluble and has been shown to exist principally as a monomer at a neutral pH and to oligomerize as the solution decreases to pH 5 [9,15]. Magnetic resonance studies of full-length channels in MSP1D1 lipid nanodiscs indicate that the C-termini of individual channels associate at a neutral pH and dissociate at pH 5 [37]. However, the lipids used in that investigation were relatively short (14 carbon acyl chain), saturated DMPC lipids, and single-component phosphocholine (PC) bilayers have vastly reduced the open probability of KcsA compared to when an anionic lipid headgroup is at least a component of the bilayer [6].

X-ray and small-angle neutron scattering data support a model where the C-termini within a tetrameric channel are close in space at a neutral pH and become much more widely separated at pH 5 [30]. Those same data suggest that the changes in conformation of the bundle of C-termini accompany a change in conformation in the transmembrane domain, but that changes to the transmembrane domain do not occur in channels with truncated C-termini.

These previous studies in aggregate suggest a putative model where the C-termini within a tetramer electrostatically coordinate to stabilize channels at a neutral pH, and at an acidic pH the bundle of C-termini become more loosely associated, leading to allostery affecting transmembrane gate conformation. This model suggests that the bundle of C-termini would be rigid at neutral pH and more dynamic at acidic pH. These putative changes in dynamics provide motivation to study the C-terminus of KcsA by hybrid solid-state solution-state NMR methods. Arguably, proteoliposomes provide a gold standard for authentic membrane environments. With that perspective in mind, we show that high-resolution magic angle spinning NMR (HR-MAS) can be utilized to probe the C-terminus of KcsA to support this model while channels are embedded in a native-like membrane environment.

## 2. Methods

### 2.1. Protein Expression, Purification, and Reconstitution

Wild-type KcsA was expressed as an N-terminal His_6_ fusion protein cloned onto a PASK90 plasmid, which confers ampicillin resistance, and was over-expressed in *Escherichia coli* cells using established protocols developed by our group [38]. 

Expression of fractional deuteration with uniform ^13^C and ^15^N KcsA was accomplished with minor modifications from [36]. Briefly, KcsA culture using Bl21(DE3) *E. coli* cells (New England Biolabs, Ipswich, MA, USA). Cells were grown at 35 °C in LB selection media to OD_600_ = 0.8 and collected by centrifugation. The cell pellet was rinsed in approximately 50 mL of D_2_O (Cambridge Isotopes, Cambridge, MA, USA). The cells were then suspended in 500 mL of M9 minimal media solution (See Supplemental Information) using 98% D_2_O. The M9 media was supplemented with 1.5 g per liter U-^13^C-glucose and 0.5 g ^15^N-ammonia. This culture was allowed to grow for 1 h at 37 °C, then induced with anhydrotetracyclin (aTC) (Sigma-Aldrich, Saint Louis, MO, USA), and allowed to express at 25 °C for 10 h. 

KcsA was purified in the same manner regardless of labelling strategy. Pellets were lysed by French Pressure cell (Thermo, Waltham, MA, USA), detergent solubilized in buffer containing decyl-β-maltopyranoside (Anatrace, Maumee, OH, USA), and purified by His-Select nickel affinity (Sigma-Aldrich, Saint Louis, MO, USA) chromatography detergent (DM).

We prepared a KcsA C-terminal truncation construct, containing only residues 1–125 (KcsA-∆125), cleaving residues 126–160, by incubating purified KcsA in detergent with bovine α-chymotrypsin (Sigma-Aldrich, Saint Louis, MO, USA) for 3 h at 35 °C. KcsA was isolated using Ni-affinity gel as described above.

Liposomes were formed in a 9:1 mass ratio of 1,2-dioleoyl-sn-glycero-3-phosphoethanolamine (DOPE) to 1,2-dioleoyl-sn-glycero-3-phospho-L-serine (DOPS) (Avanti, Alabaster, AL, USA) by solubilizing a thin film of the lipids in detergent buffer and mixing with detergent solubilized KcsA in a 1:1 lipid-to-protein mass ratio. The lipid-protein solution was dialyzed into the final buffer with three buffer changes. The presence of KcsA as a tetramer in the liposomes was verified by SDS-PAGE before and after NMR experiments.

### 2.2. NMR

*J*-coupled-based experiments were performed on a Bruker magnet with a proton field of 750 MHz using a 4 mm high-resolution magic angle spinning probe (HR-MAS) equipped with ^1^H/^13^C/^15^N/^2^H channels and a 40 G/cm gradient coil oriented along the magic angle. HSQCs were collected using the Bruker sequence hsqcetgpsi for ^13^C-resolved spectra, and hsqcetf3gpsi2 for ^15^N resolved spectra. hCCH-TOCSY spectra were collected with full-rotor period synchronized TOCSY 10 kHz spinlocks using the Bruker sequence hcchdigp3d2. HcCH-TOCSY indicates ^13^C as F2 and ^1^H as F1 and hCCH-TOCSY indicates ^13^C in both indirect dimensions. Site-specific *T*_2_ measurements were collected by adding a single rotor-synchronized ^13^C spin-echo between the two inept transfers and increasing the delay of the echo over at least five steps until magnetization had decayed by at least 90%. Quantitative ^1^H spectra were collected with calibrated ^1^H 90° (typically ~9 µs), with recycle delays of at least 5 s (the longest *T*_1_ in samples was typically ~0.9 s) with pre-saturation on the H_2_O resonance during the recycle delay and a presaturation field strength of approximately 25 Hz. Typically, 4 to 16 scans were collected to reach a signal-to-noise ratio of more than 20 for the CH_2_ region. Cross-polarization MAS was performed with a Bruker 750 Avance I spectrometer using a 3.2 mm ^1^H/^13^C/^15^N e-free probe at 16.6 kHz at 275 K rotor set temperature.

### 2.3. Sucrose Gradients

Sucrose Gradients were prepared by layering equal volumes of two sucrose concentrations (depending on upper and lower bounds of gradient desired) in buffer in 12 mL ultracentrifuge tubes (Beckman, Brea, CA, USA), then applying a preprogramed algorithm to mix the layers using a Gradient Master (BioComp Instruments, Fredericton, NB, CA) and cooling to 4 °C overnight. Proteoliposomes were prepared as described above except, a rhodamine-conjugated lipid (1,2-dioleoyl-sn-glycero-3-phosphoethanolamine-N-(lissamine rhodamine B sulfonyl) (Rhod-PE) (Avanti, Alabaster, AL, USA) was added to the lipid mixture to more easily visualize and quantify the mixtures. The lipid to protein ratio was 1:1 by mass. The lipid mixture was 900:10:1 PE-PS-Rhod PE by mass. A total of 20 mg of KcsA proteoliposomes was suspended in buffer and added to the top of the gradient. The tube was subjected to ultracentrifugation at 25,000 RPM, corresponding to a relative centrifugal field ranging from 47,200× *g* to 107,000× *g*, for 24 h at 4 °C with the slowest acceleration and no brake during deceleration. The bottom of the tube was pierced with a 20 Ga needle that was then removed and the tube was allowed to flow under gravity at 4 °C. Fractions were measured by UV-VIS at 280 nm for the presence of protein and 560 nm for the presence of lipids as a qualitative measure. To quantify protein we solubilized KcsA proteoliposomes in Triton-X100 and measured against a standard curve using bromophenol blue. We performed SDS-PAGE and a Pierce silver stain kit (ThermoFisher, Waltham, MA, USA) was used to visualize protein in gradient fractions. 

See the Supplementary Information for more detailed materials and methods.

## 3. Results

Since the C-terminus of KcsA is not resolved by CP-MAS nor by crystallography, we reasoned that the order and rigidity required by these methods is likely lacking. All regions of KcsA when channels are detergent-solubilized are accessible to solution-state NMR. However, the long rotational correlation time of channels in bilayers leads to no well-resolved resonances in traditional solution-state NMR due to anisotropic effects.

Therefore, we pursued a strategy to reduce anisotropy and increase radio field homogeneity using magic angle spinning (MAS). We made *J*-based NMR measurements, which work well on isotropic systems, to increase the likelihood of capturing signals from dynamic regions of the protein. An HR-MAS probe is ideally suited for these purposes. HR-MAS detects the ^1^H signal, and as ^1^H has much a much higher gyromagnetic ratio and typically much shorter *T*_1_ relaxation periods than ^13^C and ^15^N, detecting on ^1^H leads to practical gains of 100 times or greater signal-to-noise ratio than detecting on the heavier nuclei. Additionally, unlike CP-MAS probes, HR-MAS probes have a gradient coil, enabling modern, gradient-selected pulse sequences.

### 3.1. HR-MAS Selectively Detects Signals from the KcsA C-Terminus

**Figure 2:**
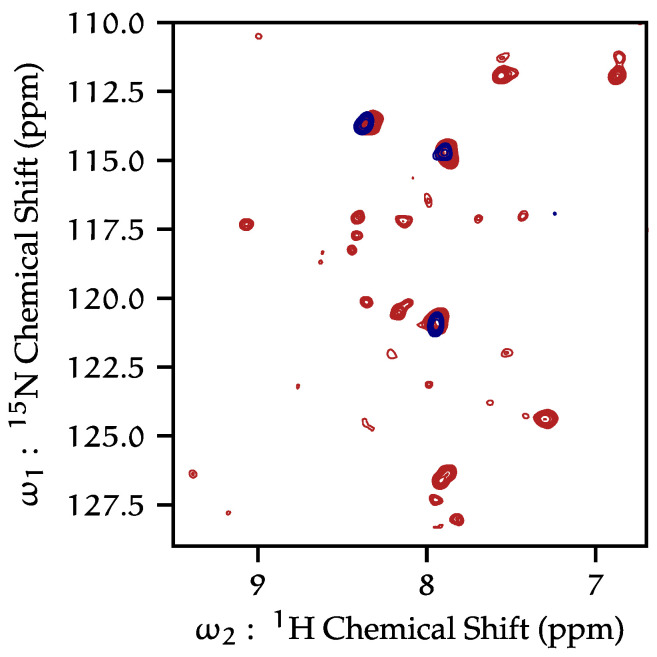
^1^H-^15^N HSQC by HR-MAS of full-length wild-type KcsA (red), and KcsA-∆125 (navy); both samples pH 7.25, 50 mM K^+^, 308 K, 5 kHz MAS.

We prepared proteoliposomes with a C-terminal truncation construct, containing only residues 1–125 (KcsA-∆125) by cleaving residues 126–160 by enzyme reaction (Supplementary Materials Figure S1). We collected both ^15^N (Figure 2) and ^13^C (Supplementary Materials Figure S2) HSCQ spectra of the KcsA-∆125 truncation mutant in liposomes and compared these to spectra of proteoliposomes containing full-length KcsA. Comparing full-length KcsA to KcsA-∆125, we conclude that much of the protein signal in spectra of the full-length construct arise from the C-terminus. The relatively few signals observed in the ^1^H-^13^C HSQC of the KcsA-∆125 sample arise mainly from the exogenous phospholipids into which the protein is reconstituted. Similarly, ^1^H-^15^N HSQC spectra (Figure 2) also show that most (though not all) resonances are eliminated when the C-terminus is truncated, indicating that HR-MAS signal from KcsA principally arises from the C-terminus. Throughout this study, SDS-PAGE was used to verify that all samples were of tetrameric KcsA before and after NMR experiments. Protein-free liposomes were examined to assign resonances arising from lipids. We do not present data of KcsA-∆125 at low pH, as each time such a sample was prepared, we detected only monomeric KcsA.

### 3.2. Cleavage of the KcsA C-Terminus Causes Conformational Heterogenity near the Selectivity Filter

**Figure 3:**
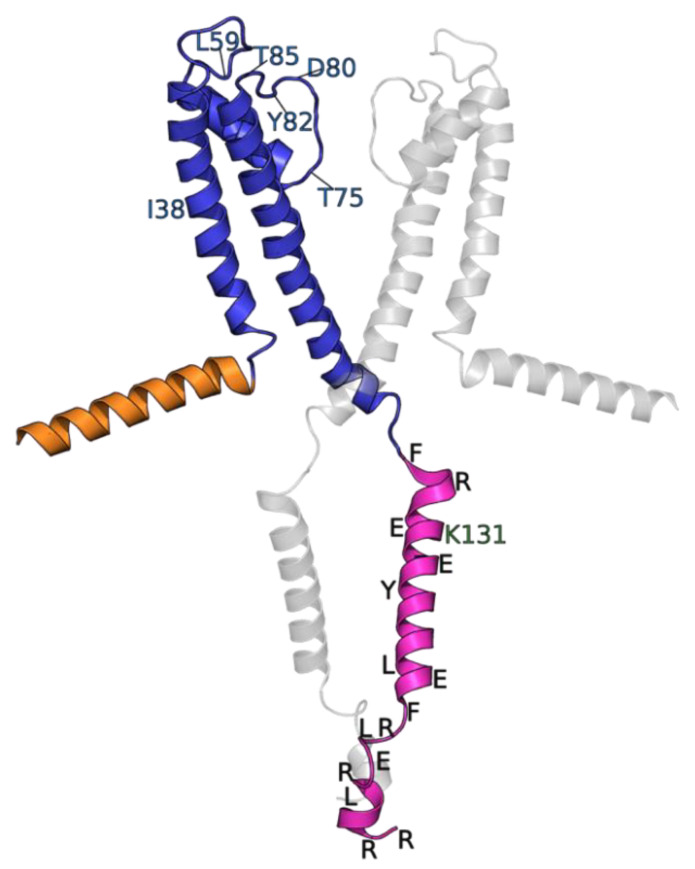
Cartoon of KcsA adapted from PDB: 1F6G [29], with two subunits hidden for clarity. Cytosolic N-terminus shown in orange (residues 1–22), transmembrane domain in blue (23–124), and cytosolic C-terminus in magenta (125–160). Resonances missing from CP-MAS ^13^C-^13^C spectrum of KcsA–∆125 are indicated with blue labels. Newly assigned Lys-131 indicated. Approximate locations of candidates for type-assigned resonances labeled in black with single letter amino acid code. Visualized with Pymol [39].

To inspect for changes to the KcsA transmembrane domain caused by cleavage of its C-terminus, we collected CP-MAS ^13^C-^13^C proton-driven spin diffusion (DARR) spectra (Supplementary Materials Figure S3). Chemical shift assignments from previous studies in our group [26] were used to assign the resonances that are present in the CP-MAS data of this study. These data indicate the KcsA-∆125 transmembrane domain is well-folded. However, several transmembrane resonances that are typically observed in full-length KcsA spectra are missing from the KcsA-∆125 sample, corresponding with the residues Ile-38, Leu-59, Thr-74, Asp-80, Tyr-82, and Thr-85. We map these absences onto a cartoon of KcsA in Figure 3.

The most likely explanation of these transmembrane residues missing from KcsA-∆125 data is that the C-terminal truncation leads to much greater conformational heterogeneity of the regions at the interface between KcsA’s loop regions and selectivity filter. Alternatively, or in addition to conformational heterogeneity, the deletion of the C-terminus may lead to increased dynamics within the selectivity filter, thereby making it less amenable to cross-polarization experiments, which rely on rigidity for efficient magnetization transfer. Previous studies have found a decreased open probability of C-terminal truncation mutants [2]. Regardless of the specific mechanism, these data provide evidence that the C-terminus is involved in stabilizing the KcsA selectivity filter and suggest a mechanism for the decreased open probability caused by truncating the C-terminus [6,31,32].

### 3.3. Low pH Leads to More Dynamics of the C-Terminus of KcsA Embedded into Liposomes

**Figure 4:**
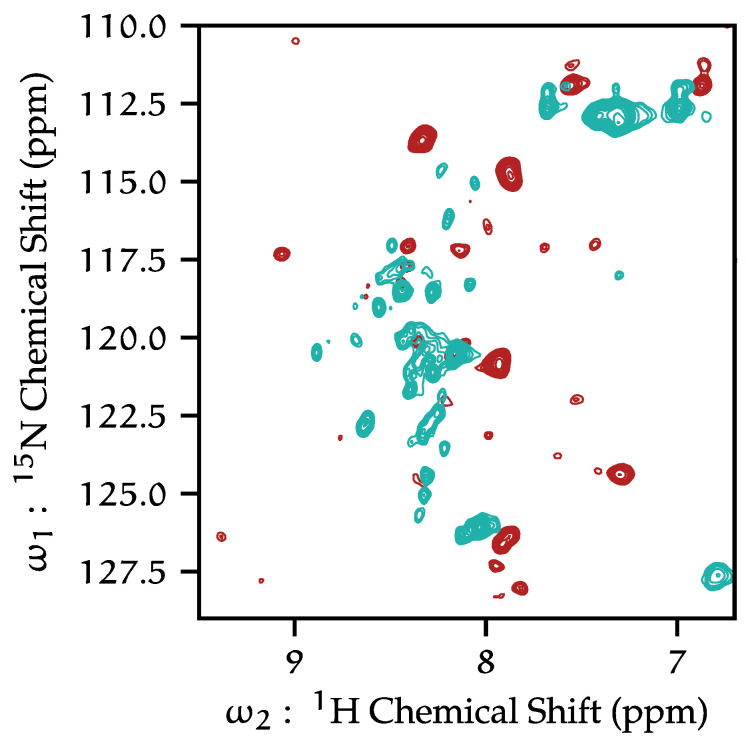
^1^H-^15^N HSQC of protonated KcsA at pH 7.25 (red), and fractionally deuterated KcsA at pH 4.0 (turquoise) by HR-MAS. 5 kHz MAS, 308 K, 50 mM K^+^.

Both ^1^H-^13^C and ^1^H-^15^N HSQC HR-MAS spectra of KcsA in liposomes at neutral pH contain few resonances, most of which originate from the C-terminus. When the pH of the sample is lowered to pH 4, both the number and the distribution of resonances in spectra increases. Figure 4 presents an example. The dramatic increase in the number of resolved resonances and an overall increase in signal strength suggest that KcsA is undergoing much greater conformational dynamics at a low pH. The ^13^C and ^15^N chemical shifts of resonances that appear in our HR-MAS spectra do not correspond with shifts appearing in our solid-state spectra. Additionally, we have demonstrated in previous studies that there is no comparable reduction in peaks in CP-MAS spectra when moving from pH 7 to pH 4 [26]. Thus, we conclude these previously unresolved resonances arise from KcsA termini. We were able to collect a variety of 3D correlations (HNCO, HNCA, HcCH-TOCSY, and hCCH-TOCSY). We are unable to unambiguously assign resonances in ^15^N correlation spectra by residue or by type, but we are able to make assignments in our ^13^C data, and those details are given in Section 3.7.

### 3.4. Leucine as a C-Terminal Conformational Indicator

**Figure 5:**
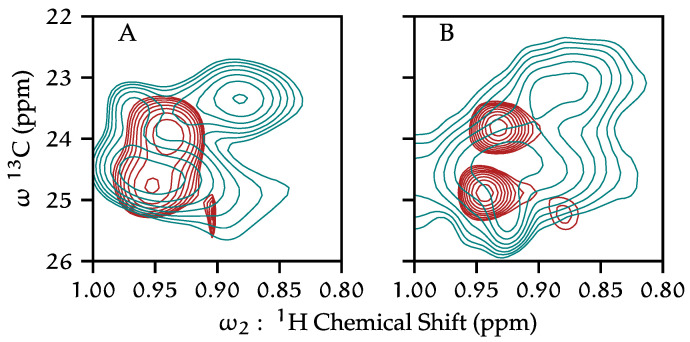
^1^H-^13^C HSQC spectra of leucine Hδ of KcsA in liposomes. pH 7.25 (red contours) and pH 4.0 (turquoise contours) are shown, with fully protonated KcsA samples (**A**) and fractionally deuterated KcsA samples (**B**).

The conformational and dynamical changes that the protein undergoes from neutral to low pH can be observed in ^1^H-^13^C HSQC data. This is convenient, as a high-quality ^1^H-^13^C HSQC can be collected in approximately three hours, where a ^1^H^15^N HSQC with similar signal to noise requires approximately 16 h. We find the leucine ^1^H-^13^Cδ (methyl) correlations in the HSQC to be a reliable indicator of state. We made the residue type assignment from characteristic shifts of connected spin systems from 3D TOCSY data (see Section 3.7 for more shift assignments). The number and chemical shifts of peaks in HSQC data are replicable at both neutral and acidic pH, reflecting systematic changes to the entire spectrum, and to the pair of leucine in particular (Figure 5). We cannot confirm whether we are detecting the same leucine residues in the sequence at each pH condition. These leucine resonances are not present in the C-terminal truncation construct, confirming that these resonances arise from the C-terminus, which has a total of three leucine residues (Leu-144, Leu-151, Leu-155). The difference in the shift dispersion (line broadening) at a low pH is a strong indication that the conformation of the C-terminus is more heterogeneous than the conformation at neutral pH. These observations support a model where the KcsA C-termini at neutral pH are relatively well-defined, and the termini bundle becomes more heterogeneous at low pH.

### 3.5. ^13^C T_2_ Relaxation

We sought to estimate the relative anisotropy of resonances using site-specific ^13^C transverse relaxation of the protein signal of uniformly ^1^H, ^13^C, and ^15^N-labelled KcsA in liposomes. We collected HSQC data with a variable-length dephasing period before *T*_2_ evolution (Supplementary Materials Table S1: ^13^C *T*_2_s). The average protein ^13^C *T*_2_ relaxation rate was 2.9 ms, and the average for lipid headgroup ^13^C was 5.2 ms at 308 K, 9 kHz MAS, and 10 kHz heteronuclear decoupling. The optimal transfer time for a single Cα-N polarization transfer for the refocused-INEPT is expected to be 45 to 70 ms [40]. Our relaxation data predict a maximum theoretical signal yield of only 4% when compared to yield possible in the absence of relaxation, as predicted from the density function of the refocused INEPT experiment, $\Gamma \left(t\right)=sin\left(\pi J_{N-C\alpha }t\right)exp\left(\frac{t}{T_{2}}\right)\;$ [41]. Under these conditions, even the highest-sensitivity ^1^H-detected backbone experiments, such as the HNCaH and HNCO (out-and-back), which require multiple INEPT transfers, are not feasible with fully protonated samples.

### 3.6. Fractional Deuteration

**Figure 6:**
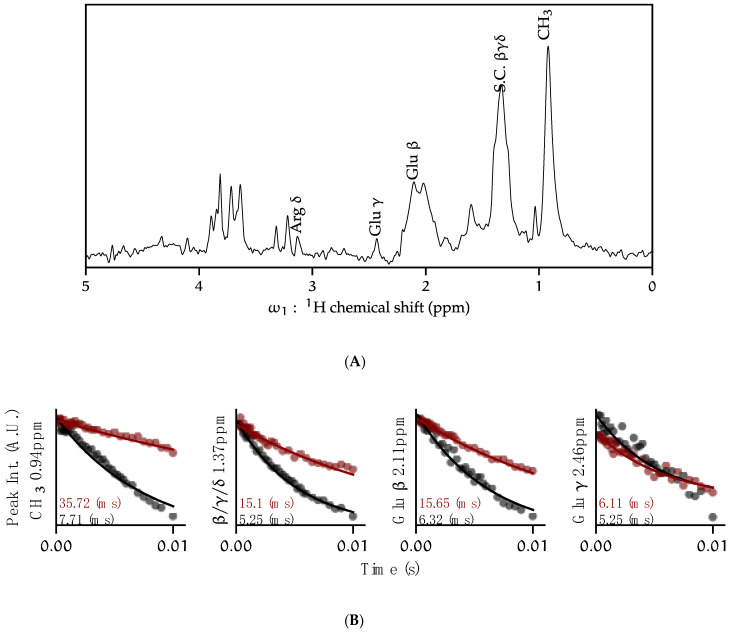
The *T*_2_ relaxation time is lengthened due to fractional deuteration of KcsA. (**A**) ^1^H-^13^C double-INEPT (1D refocused HSQC) spectrum of U-^1^H, ^13^C, ^15^N-KcsA in proteoliposomes (9:1 DOPE-DOPS) with peak annotations. Assignments are based on 2D and 3D data. (**B**) ^13^C relaxation of selected peaks from 1D double-INEPT for fully protonated KcsA (black), and fractionally deuterated KcsA (red). Both samples U-^13^C,^15^N at pH 4, 50 mM K^+^, 9 kHz MAS, 308 K.

Anisotropic interactions, particularly dipolar couplings, lead to shorter *T*_2_ relaxation values for proteins. Residual dipolar couplings with ^1^H nuclei vastly attenuate *J*-based coherence transfer in backbone experiments. Replacing ^1^H with ^2^H in proteins is a well-established method of reducing these dipolar couplings, thus increasing *T*_2_ times and improving resolution and coherence transfer efficiency [42,43]. Although reports of high [5,11,37] and perdeuterated [12] KcsA exist in the literature, the expression of perdeuterated KcsA led to poor yields in our research. We followed previous work that found that expressing proteins in D_2_O minimal media supplemented with U-^1^H, ^13^C-glucose and ^15^N-ammonium chloride leads to proteins that have very high levels of deuteration at the H-Cα position and fractional deuteration in the sidechains that vary by amino acid [35,44]. Here, we expressed KcsA in this manner and incorporated it into liposomes. ^13^C *T*_2_ relaxation profiles were determined using a 1D back-INEPT experiment with a dephasing period. We present profiles for the methyl region, the aliphatic region, Glu-^13^Cβ, and Glu-^13^Cγ as these are readily identified on the 1D spectrum of our samples (Figure 6). The results show that the relaxation profile is vastly improved in fractionally deuterated samples over uniformly protonated samples. The Glu-Cγ profiles are similar for both samples, which agrees well with previous observations that both glutamate γ protons tend to remain protonated in this labeling scheme, and a portion of the Glu-Hβ remains protonated in this labeling scheme [35]. With the fully protonated sample, signal was insufficient to collect 2D HNCA or HNCO correlation experiments, while with fractional ^2^H-KcsA, full 3D datasets were obtained, showing 13 well-resolved resonances (Supplementary Materials Figures S8 and S9). Yet, these improvements still leave backbone walk experiments out of reach.

### 3.7. Chemical Shift Data

**Figure 7:**
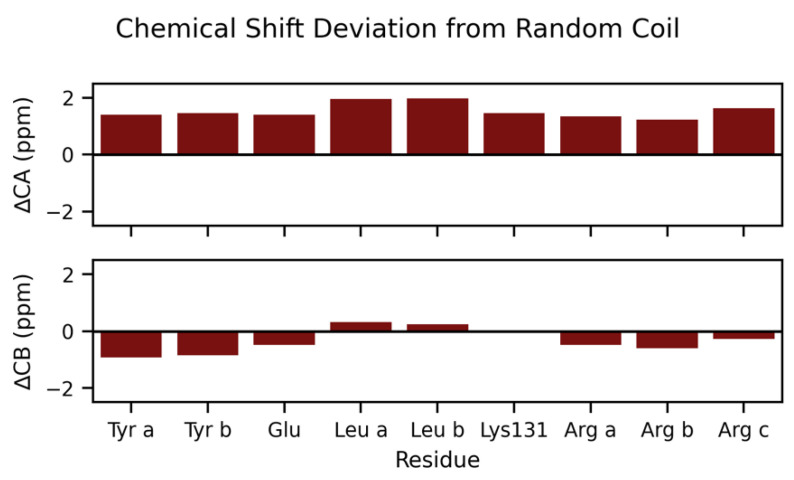
Chemical shift deviations from random coil of residues in the KcsA C-terminus at neutral pH condition KcsA using hCCH-TOCSY data, U-^1^H, ^13^C, ^15^N-KcsA in proteoliposomes (9:1 DOPE-DOPS) at pH 7.2, 50 mM K^+^.

Table 1 provides chemical shifts of spin systems identified in 3D hCCH-TOCSY data of U-^1^H,^13^C,^15^N-KcsA in proteoliposomes at pH 7.2. Spin systems were type assigned based on characteristic chemical shifts. Lys-131 is assigned based on it being the only lysine residue in the C-terminus and that spin system is absent in KcsA-∆125 spectra. The deviation from random coil of type-assigned ^13^Cα resonances from Table 1 are plotted in Figure 7.

### 3.8. Lipid Stability in NMR Samples

Solid-state NMR samples routinely spend a week or more in the probe. We made near-complete ^1^H and ^13^C assignments of lipids (except highly degenerate resonances in the aliphatic chain) in KcsA proteoliposome samples (Table 2) using hCCH-TOCSY experiments (Supplementary Materials Figure S7). At least six resonances frequently appeared in KcsA proteoliposome data that do not correspond to known lipid species or that are consistent with protein chemical shifts. Chemical shifts of correlated spin systems correspond to the literature values of free glycerol and free ethanolamine [45,46]. Neither glycerol nor ethanolamine are used in the preparation of our samples, and the signal strength from these compounds is too high to suggest trace contaminants. Chemical logic suggests that these components result from degradation of the lipids. The chemical shifts of degradation products are documented in Table 2, and their presence (or absence) in various samples is further with additional detail is in Supplementary Materials Tables S2 and S3. We and others have previously reported that exogenous lipids co-purify with KcsA expressed in *E. coli* containing a phosphoglycerol headgroup [22]. The copurified lipids can be identified because they are expected (like the purified protein) to have uniform enrichment of ^13^C and ^15^N, which is in contrast to exogenously lipids that are added during reconstitution, which would have natural abundance of ^13^C and ^15^N content. The most plausible source of ethanolamine is from hydrolyzed exogenous PE lipid headgroups. We have not identified the catalyst for this hydrolysis. In our samples, HSQC signal for free glycerol is invariably much greater than that of free ethanolamine (when ethanolamine is detectable at all). These data suggest to us that at least some of the glycerol arises from hydrolysis of co-purifying ^13^C-enriched PG. Phosphoglycerol or free glycerol signal is present in nearly all KcsA samples, with most containing signals from both.

**Table 2:** Chemical shift assignments for liposome lipids and lipid hydrolysis products measured in this paper at pH 4–7, 50 mM KCl, 308 K, 5 kHz MAS. See Figure 8 for nomenclature.

Site	^1^H (ppm)	STD	^13^C (ppm)	STD
Fatty acid 12 (ω)	0.88	0.018	16.6	0.12
Fatty acid 11 (ω − 1)	1.3	0.017	25.3	0.06
Fatty acid 8 (CH2-HC=C)	2.02	0.038	29.9	0.19
Fatty acid 6 (CH2)	1.29	0.022	32.1	0.1
Fatty acid 10 (ω − 2)	1.27	0.02	34.6	0.08
Fatty acid 4 (α-CH2)	2.33	0.033	36.6	0.24
PE β	3.24	0.037	43.3	0.37
Ethanolamine β	3.19	0.088	44.2	0.1
Ethanolamine α	3.84	0.095	60.3	0.2
PE α	4.11	0.027	64.6	0.48
PG γ + glycerol a	3.67	0.025	65.4	0.1
PG γ + glycerol b	3.61	0.044	65.4	0.09
G3 a	4.23	0.028	67	2.2
G3 b	4.46	0.027	67	2.3
G1	4.3	0.23	67.5	3.4
PG α a	3.94	0.022	70	1.8
PG α b	3.88	0.025	70	1.8
PG β	3.93	0.028	72	2.2
G2	5.26	0.012	73.3	0.15
glycerol (CH1)	3.83	0.035	74	2.4
Fatty Acid 9 (HC=C)	5.31	0.027	132.2	0.12

**Figure 9:**
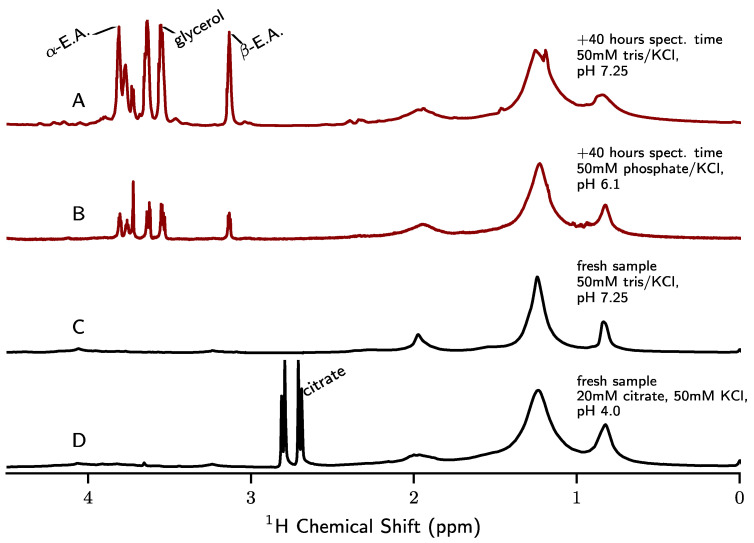
Quantitative ^1^H direct-excitation MAS spectra (with water presaturation) of KcsA proteoliposome samples. Samples that contained free ethanolamine signal are displayed in red (A and B). The samples that contain only PE (intact lipid) and no ethanolamine are displayed in black (C and D). All spectra were collected at 308 K, 5 kHz MAS. Spectra are normalized to the bulk CH_2_ signal (∼1.2 ppm). All samples are U-^1^H-^13^C-^15^N-KcsA in 9:1 DOPE liposomes, LPR = 1, by mass with additional details shown in the figure. Free glycerol, ethanolamine (E.A.), and citrate peaks are labeled.

In Figure 9, we demonstrate that a direct polarization ^1^H experiment with pre-saturation conducted at 308 K in an MAS probe is sufficient to diagnose the presence of lipid hydrolysis products. Although the spectra we display were collected in an HR-MAS probe, we also find that this experiment can be accomplished effectively in an ^1^H/^13^C/^15^N e-free CP-MAS probe configured for ^1^H detection. The sharp peaks arising from mobile solutes of lipid hydrolysis were identified by chemical shift and confirmed through ^1^H-^13^C HSQC and hCCH-TOCSY data (see Supplementary Materials Figure S4 for example assignment data). The principal small molecule (sharp-peaked) signal is from buffer components and water-soluble hydrolysis products of lipids such as glycerol and ethanolamine.

We took a survey of preserved samples from previous studies that used KcsA liposomes from our group and for each, we investigated the presence of lipid hydrolysis product by ^1^H-^13^C HR-MAS HSQC. The spectra were examined for the presence of PG, ethanolamine, and glycerol, and the chemical shifts of these compounds were then used to assign peaks in quantitative ^1^H spectra of the same samples. The results are summarized in Supplementary Materials Table S2. The shifts from HSQC data were used to assign the peaks in the ^1^H data, and thereby the presence or absence of the glycerol and ethanolamine was verified.

### 3.9. Effect of MAS Centrifugation on Samples

Magic angle spinning places samples under intense centrifugal forces. So, we conducted experiments to attempt to mitigate these forces. The centrifugal forces on the sample during MAS are described by:

(1) $$RCF=4.025\times 10^{-3}rQ^{2}$$

where *RCF* is given in *g*-force (g), *r* is the radius from the center of the rotor in millimeters and *Q* the spinning frequency in Hz, wherein sample fractions at the rotor wall experience the greatest forces.

We hypothesized that varying MAS might lead to changes in protein structure that could be identified in spectra. To test this, we compared ^1^H-^13^C HSQC spectra of KcsA in liposomes at 5 kHz versus 15 kHz MAS, increasing the RFC by a factor of 9 at the rotor wall, but did not identify any systematic differences. Further, we have found MAS speeds more than 10 kHz for these samples lead to rotor crashes, whereas we had no such difficulty at 5 kHz.

As an alternative, we sought to balance centrifugal forces with buoyant forces by density matching the sample to the buffer. We pelleted KcsA following dialysis, and then subjected the proteoliposomes to 25 rounds of freeze–thaw using liquid nitrogen and a 30 °C water bath. This led to the formation of proteoliposomes of unilamellar as well as multilamellar or oligomellar structures (Supplementary Materials Figure S6) similar to previous reports [47,48]. To characterize the proteoliposomes resulting from this freeze–thaw procedure, we loaded the samples onto a buffer and uniform sucrose gradient (5–60%) and performed isopycnic ultracentrifugation. In order to visualize the proteoliposomes on the column and quantify the lipid concentration, we included a rhoadamine-congated lipid (1,2-dioleoyl-sn-glycero-3-phosphoethanolamine-N-(lissamine rhodamine B sulfonyl) (Rhod PE) in the lipid mixture. The lipid mixture had a mass ratio of 900:10:1 PE-PS-Rhod PE, and the total lipid to protein mass ratio was 1:1.

Following centrifugation, aliquots were taken, and portions of each aliquot were used to determine protein concentration by solubilizing proteoliposomes with a solution of Triton-X100 and bromophenyl blue and comparing UV-VIS absorbance at 610 nm with a standard curve [49]. Total lipids were extracted from aliquots using chloroform and methanol [50] and quantified using a standard curve based on UV-VIS absorbance of Rhod-PE at 560 nm. Density of aliquots was determined by analytical balance and calibrated micropipette using low retention tips.

The isopycnic gradient experiment reveals distinct sets of populations distinguished by their densities (Table 3). All the data described in this paper (and many other studies) would presumably contain both of these populations as we were unable to efficiently separate these populations.

We hypothesized that placing liposomes in a density-matched matrix would diminish the deleterious mechanical effects of magic angle spinning. However, despite many repeated attempts at isopycnic preparations, we were unable to achieve total protein concentrations of greater than 5% in aliquots. As NMR is a relatively insensitive technique, and therefore sample concentration is a critical feature of obtaining strong signal in a reasonable amount of time, we sought alternative methods to density-match the sample.

**Figure 10:**
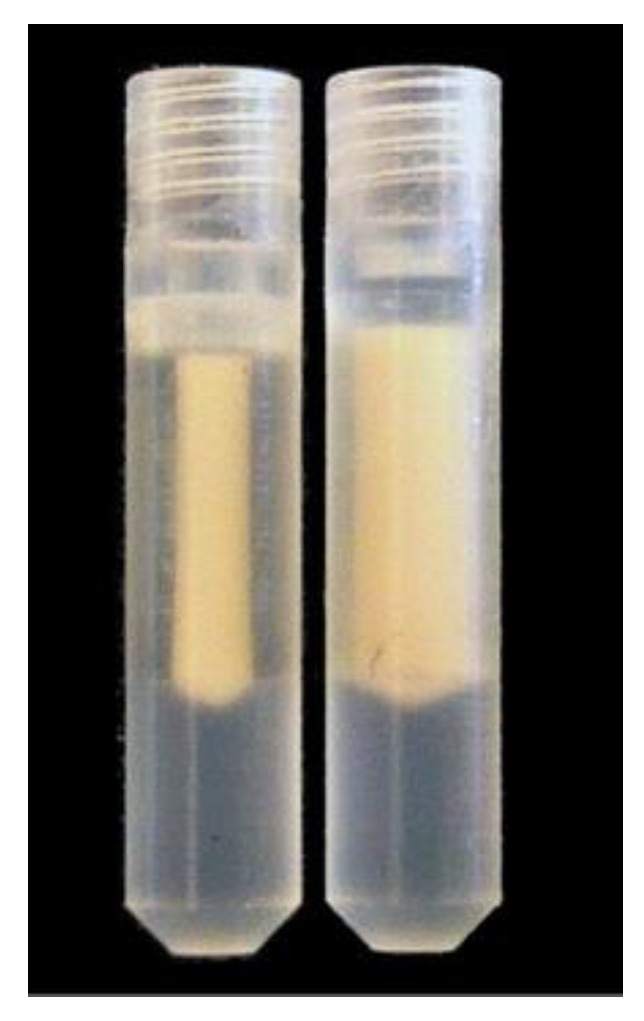
KcsA proteoliposomes in MAS rotor inserts post-experiment. Left: ‘floating’ sample in sucrose-augmented buffer, and Right: ‘sinking’ sample with no sucrose in buffer.

MAS can be expected to exert pressure on the proteoliposomes, potentially changing their morphology or hydration, and therefore affect both function and NMR detection. To reduce net force on the proteoliposomes, we sought to prepare an isopycnic solution of the proteoliposomes. To roughly match the density of the KcsA pellet to the buffer, we ‘floated’ the unilamellar proteoliposomes pellet by adding aliquots of 60% *w*/*w* sucrose-augmented buffer to a tube containing the pellet, then vortexed and centrifuged in a bench-top centrifuge and observed whether the pellet sank or floated. We found that this process can be reversed (i.e., cause a floating pellet to sink) by adding an aliquot of buffer without sucrose to the tube. The process can be repeated in either direction indefinitely. Thus, this experiment was dubbed a reversible ‘elevator’ experiment and provided a means to titrate the density to match KcsA proteoliposomes. Because we used transparent rotor inserts, we were able to verify that the floating pellet does indeed migrate to the center of the rotor during MAS (Figure 10). From the observation that the sample floats in a high-sucrose background, we assert that under these conditions, the sample experiences greater buoyant force than centrifugal force. Further, the floating sample migrates to the center of the rotor, where *g-*forces diminish to zero. We estimate the maximum radius of a pelleted sample at 0.96 mm (the inner diameter of the rotor insert) and the maximum radius of a floating sample at 0.63 mm (Supplementary Materials Figure S11), reducing the maximum centrifugal force by a factor of 0.66.

We did not identify dramatic differences in ^1^H-^13^C or ^1^H-^15^N HSQC by HR-MAS spectra of KcsA unilamellar proteoliposomes that were ‘floating’ versus pelleted (data not shown). Cryo-electron microscopy (Supplementary Materials Figure S6) shows that before MAS, many small unilamellar vesicles are present. Following MAS, no unilamellar vesicles could be found. Instead, post-MAS vesicles in pelleted and floating samples are less well-ordered and show multilamellar, oligolamellar, and aggregated morphology. This suggests that the floating samples are not protected from changes to lipid morphology under MAS conditions.

## 4. Discussion

We present evidence that KcsA in model liposomes assumes distinct conformations at pH 7.25, where the channel is inactive, and at pH 4.0, under which conditions the channels can activate. Here, we use the same lipid system used previously to model KcsA activation and inactivation states [25] and allosteric coupling between sites within KcsA [3,26]. Thus, sample conditions in this study are appropriate for studies that connect channel structure to channel function.

The conformational switch presented here is consistent with previously proposed structural models. A sequential spin-labeled ESR structure of KcsA in bilayers indicated significant mobility of the C-terminus at pH 4 [29]. On the other hand, full-length KcsA with its C-terminus stabilized by Fab antibodies at neutral pH was rigid enough to capture using X-ray crystallography [1].

This study and others [37,51] report two distinct dynamical regimes of the KcsA C- terminus as a function of pH. Dramatic changes can be observed in the ^1^H-^15^N HSQC data (Figure 2), with many more peaks with typically much narrower line widths at low pH compared to neutral pH. This is particularly clear when comparing fractionally deuterated samples but is also observed with fully protonated samples, showing reproducibility and independence of the labeling scheme. These data indicate that as the pH is lowered, the KcsA the C-terminus moves more freely.

Our analysis of hCCH-TOCSY spectra resolved ten groups of peaks that can be assigned by amino acid type (summarized in Table 1). These data alone established an additional new site-specific spectral assignment (Lys-131). We also identify three arginine, two leucine, two aromatic (Phe or Tyr), and glutamic acid spin systems. We are unable to definitively distinguish between Phe/Tyr, though Tyr is more likely than the respective alternative based on canonical chemical shift data. All these spin systems are absent in spectra of KcsA-∆125. So, the resonances are almost certainly from the C-terminus. Previous X-ray structures of full-length KcsA in the closed confirmation [1] and whose data agree with EPR structural data [29] suggest that, in the C-terminus, Phe-125, Arg-127, Lys-131, Glu-135, Arg-139, Arg-142, Glu-146, Arg-159, Arg-160 are solvent-exposed and are therefore most likely to have resolved side-chain resonances. The most compact sequence of amino acids that conforms to the evidence would be from Phe-125 to Leu-151 (FVRHSEKAAEEAYTRTTRALHERFDRL) (see Figure 3 for a visualization of these potential sites). Even within this short sequence, several arginine and glutamic acid residues would be unresolved amongst the amino acids we have type-assigned. According to the previous structural models, all the candidate sites for hydrophilic residues (Lys, Arg X3, Glu X2) are on the solvent-exposed surface, whereas the leucine side chains are likely to be facing other protein subunits and the phenylalanine and tyrosine.

The ^13^Cα resonances of type-assigned residues have systematically higher shift values than mean values for random coil (Figure 7), suggesting that the residues we detect are likely to be in helical arrangement [52]. Several other studies have examined chemical shift deviations of KcsA, typically finding that the protein has particularly elevated shift values (often > 6 ppm) for tetramers in detergent micelles [12] and similar behavior in the C-terminal domain alone solubilized in water when it spontaneously tetramerizes at a high concentration [9]. Both those studies, however, also find small regions within the C-terminus at neutral pH that have less dramatic shift elevations (+1–2 ppm), which are putatively characterized as more dynamic, less rigid helices. Crystal structures [1,29] and EPR [53] measurements of KcsA in lipid mimics at neutral pH further support the hypothesis of the C-terminus existing as series of linked helices connected by more dynamic regions. Our study, conducted in the lipid bilayer, comports with this structural model. Specifically, it is likely that the resonances we detect are from the more mobile portions and that the most rigid portions of the C-terminus in the tightest helices remain unresolved under our experimental conditions.

This work identifies leucine ^1^H-^13^Cδ HSQC peaks that provide markers for the low versus high pH states (Figure 5). Specifically, we reproducibly observe a set of sharp leucine peaks at neutral pH and at low pH more peaks that are generally broader. This same behavior has been previously observed of the C-terminal domain alone solubilized in water [9]. EPR measurements in particular [53] suggest thatLeu-151 undergoes significant conformational rearrangement when the channel goes from a neutral-pH to a low-pH environment. We resolve additional leucine resonances at low pH, underscoring our conclusions that the KcsA C-terminus becomes more mobile at low pH. Yet, it is not clear why in our data the leucines in the C-terminus show the opposite trend of other resonances in becoming broader. These data cannot distinguish if the apparent line broadening is due to relaxation effects or due to conformational heterogeneity. Regardless, these findings support the use of leucine as markers for high-pH and low-pH conditions.

The data presented here show that HR-MAS is a viable method to investigate the structure of KcsA’s C-terminal domain. There are several interesting functional questions ready to be addressed with this system. For example, from previous studies we know that depleting the system of K^+^ in the low pH state leads to structural changes and subsequently to the inactivation of KcsA with a strong allosteric connection between binding of potassium at the selectivity filter and protonation of pH sensors at Glu-118 and Glu-120 [3]. HR-MAS could detect whether the C-terminus also has an allosterically induced conformational change, for example reverting to the more rigid structure upon depletion of K^+^ from the selectivity filter.

### 4.1. Considerations for Lipid Model Systems

In these pages, we demonstrate that ^1^H-detected NMR can be conducted on KcsA embedded in proteoliposomes, a highly cell-like model system, whereas previous ^1^H-detected studies of KcsA have been performed in model systems somewhat more removed from a native-like environment. Lipid–protein nanodiscs have shown promise for the study of KcsA. Work on KcsA in PC nanodiscs showed that the C-terminus has a pH-dependent conformation, which this work supports in 9:1 DOPE-DOPS liposomes [37]. This suggests that the lipid milieu does not determine the C-terminal helix bundle dissociation at low pH. One suggested function of the C-terminus helix bundling is that the contacts made across monomers may aid the stabilization of the KcsA tetramer at neutral pH [51]. Those data were collected on a solution-NMR instrument and found that resonances were too broad and the *T*_2_ values too short to conduct 3D backbone experiments. Here, we have demonstrated the ability to conduct 3D experiments in liposomes, and we might see significant increases in resolution and improvements in *T*_2_ relaxation by combining both nanodiscs and HR-MAS. Here, proteoliposomes are not expected to be rapidly tumbling. Instead, the protein diffusion through the lipid bilayer is the largest source of translational motion for the entire protein, and then individual domains, such as the C-terminus, are likely to undergo further localized movement that can decrease anisotropic interactions. Lipid composition varies the rate at which lipids and proteins diffuse through the membrane surface. The lipid composition and the protein-to-lipid ratio could be tuned to produce more rapid diffusion, which might lead to improved relaxation and resolution characteristics. Lateral diffusion of lipid probes is inversely proportional to bilayer thickness [54], so shorter chains might prove helpful. Lipid hydration is very important in determining diffusion, with maximum diffusion rates occurring above 40 percent water by mass [55]. Lipid acyl chain composition changes the diffusion rates, with DPPC (saturated lipid with 16 carbon chain), diffusing at more than two times the rate of DOPC (single cis-double bond per chain and 18 carbon chains) [56]. Headgroups play a dramatic role as well, with DOPG diffusing at twice the rate of DOPC [54], and single-component PG membranes have been shown to produce functioning channels. The rate of protein diffusion is not only lipid-dependent but also protein-dependent [54], and there is no good framework, of which we are aware, to predict the rate of diffusion of a particular protein in a mixture of lipids. Therefore, a lipid screen might be able to optimize the rate of diffusion of KcsA in the membrane. In this study, we used a lipid mixture selected here to previously connect structure and function. Future lipid surveys would be wise to begin with lipids shown to support active channels.

### 4.2. Effect of Magic Angle Spinning on Samples

Many protocols commonly used during MAS studies, including strong radio frequency (RF) irradiation for spectral decoupling and excessive *g*-forces from high spin rates, may be detrimental to proteoliposomes. Biophysical studies of membrane proteins operate on the assumption that the lipid mixture of the model systems is well-defined and does not change over the course of an experiment. The availability and distribution of conformational states may conceivably be affected by these forces. The bulk properties of the lipids, such as viscosity, lateral pressure, and other factors that affect the ability of protein to diffuse through the bilayer, are also likely to be influenced by these experimental conditions. Organic solids such as proteoliposomes frequently have dense networks of protons, where neighboring proton pairs typically have couplings more than 30 kHz. Therefore, experiments often apply MAS rates and RF fields to achieve 100 kHz or more of decoupling to obtain narrow lines, applying extreme centrifugal forces on samples.

For example, 3.2 mm rotors typically have a maximum safe spinning frequency of 24 kHz and an inner diameter of 2.17 mm, meaning at top frequency, objects at the rotor wall (from Equation (1) above) experience a relative centrifugal force of 5.0 × 10^6^× *g.* The most advanced equipment can result in substantially stronger forces. Even relatively slow spinning frequencies are known to damage biological tissue. Lipid-laden mouse adipocytes experience nearly 20% lysis after 2 h of MAS at 23× *g* [57]. Spinning at 20× *g* for 1 h substantially alters human prostate tissue morphology [48]. Under our experimental conditions, proteoliposomes migrate to the rotor wall during MAS, effectively pelleting samples.

Isopycnic sample preparation by sucrose gradient ultracentrifugation led to dilute proteoliposome samples in a high concentration of sucrose, which contributes significant background signal to spectra. These are obviously suboptimal conditions for NMR. While not useful for spectroscopy, these experiments did uncover a bimodal distribution of proteoliposome densities. This finding underscores a major theme throughout these studies: the typical methods used to prepare proteoliposomes for NMR leads to samples that are of heterogenous nature, and solid-state NMR samples deserve more scrutiny by complimentary biophysical techniques.

The alternative method of adding highly concentrated sucrose to samples to make the buffer slightly denser than the proteoliposomes to form a “floating pellet” led to a scenario in which the liposomes migrate to the region where the least amount of centrifugal force is present, namely the center of the rotor, where the *g*-force approaches zero. This allowed for much higher sample concentrations, but with still significantly less sample than a fully packed rotor and with significant small molecule background signal. The lack of dramatic changes to spectra does not justify the use of this technique routinely. However, the slight perturbations we observe suggest that this topic may warrant further investigation.

These studies reiterate our group’s previously reported findings that the structure of proteoliposome samples is heterogeneous and poorly defined. Future investigations into the effect of magic angle spinning on sample structure would likely benefit from an alternative lipid selection. We have shown that proteoliposomes with DOPC (phosphocholine) headgroups form more predictably spherical structures that would be a better candidate for future investigations [22].

### 4.3. Lipid Degradation of KcsA-Proteoliposomes

This work supports previous findings [58] that fatty acid esters can be readily hydrolyzed under certain conditions. We have observed that on the benchtop in buffer at neutral pH, DOPE and DOPS mixtures appear to be unreacted after two months of incubation. It was surprising to find evidence of hydrolysis of fatty acid chains and lipid headgroups in proteoliposome samples with this lipid composition. In one sample, we analyzed that for samples prepared for past experiments, the lipid head groups appear to be entirely hydrolyzed. The catalytic agent leading to hydrolysis in proteoliposome samples has not yet been identified.

Free glycerol ^1^H NMR peaks for the hydrolyzed samples are of the same magnitude as the peaks for free ethanolamine, suggesting that the glycerol peaks do not arise from the co-purifying lipids, but rather from the lipids added for reconstitution of liposomes. The most likely cause of the intense glycerol peaks in the ^1^H spectra is that the lipid glycerol backbone is completely hydrolyzed from the phosphate and the fatty acid moieties. Many samples do not show any evidence for free glycerol but have signals consistent with phosphoglycerol lipid. From this, we can conclude that ^13^C-enriched PG co-purifies intact with KcsA in most cases we examined.

This study suggests that proteopliposome samples demand routine quality validation measurements, particularly for studies of the effect of lipid composition. HR-MAS requires specialized equipment and the data acquisition time for an ^1^H-^13^C HSQC is at least 3 h, making this possible but somewhat onerous. However, here we show that a 1D proton spectrum is adequate to diagnosis the presence of hydrolyzed head groups. Such a procedure requires no (or very little) change to a CP-MAS experimental setup and can be collected in a matter of minutes.

## 5. Conclusions and Perspective

For many membrane proteins, structure and dynamics information on functionally crucial dynamic loops and termini is lacking. This study demonstrated the use of hybrid solution- and solid-state NMR methods to identify signals for mobile segments of an intrinsic membrane protein in proteoliposomes under functionally relevant conditions. We documented changes in dynamics of the C-terminus upon pH-triggered activation. We show that cleavage of the C-terminus has a destabilizing effect on distant residues in loops near the selectivity filter. We also show evidence that under typical experimental conditions, lipids in proteoliposomes degrade into small molecules with sharp resonances and demonstrate fast and practical NMR experiments that can detect the presence of degradation products.

## Figures and Tables

**Figure 8:**
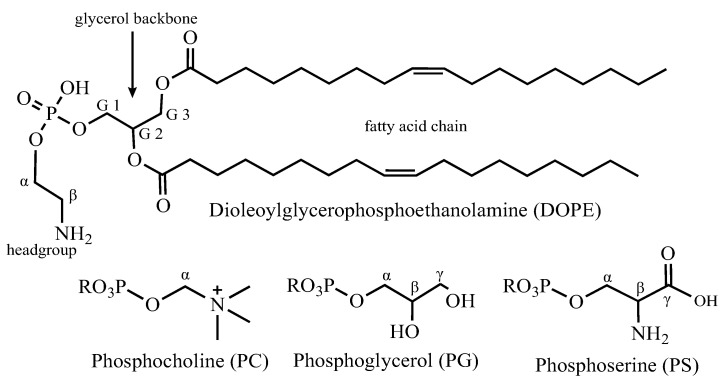
Structure of lipids of interest to this study and their nomenclature.

**Table 1:** Amino acid types identified in hCCH-TOCSY experiment of KcsA in 9:1 DOPE-DOPS at pH 7.2.

Residue	Cα	Cβ	Cγ	Cδ1	Cδ2	Cg	Hα	Hβ1	Hβ2	Hγ	Hδ
Lys 131	57.5	32.6				42.1	3.83				
Arg a	57.1	30.4	25.6	41.8			3.86	2.09		1.63	3.15
Arg b	57.0	30.2	25.6	41.8			3.83	2.06		1.63	3.14
Arg c	57.4	30.6	27.8	42.1			3.77	1.96		1.62	3.14
Leu a	56.3	42.7		23.7	24.9		3.81	1.82	1.76		
Leu b	56.3	42.6		23.8	24.8		3.81	1.82	1.76	1.82	
Phe/Tyr a	58.9	38.3					4.01	3.28			
Phe/Tyr b	58.9	38.4					4.02	3.13			
Glu a	57.5	29.7	36.4				3.83	2.12		2.44	
Glu b	57.1	29.6	36.4				4.08	2.20		2.44	

**Table 3:** Fractions of KcsA and DOPE–DOPS–Rhod-PE proteoliposomes from isopycnic sucrose gradient ultracentrifugation.

Aliquot Density (g/cm^3^)	Lipid: Protein	Proportion of Loaded Protein (%)
1.02 ± 0.01	7 ± 1	20 ± 10
1.07 ± 0.01	0.8 ± 0.1	70 ± 10

## Data Availability

NMR data and pulse sequences are available at https://comdnmr.nysbc.org/comd-nmr-dissem, (accessed on 27 June 2022).

## Supplementary Materials

The following are available online at https://www.mdpi.com/article/10.3390/biom12081122/s1, Figure S1: SDS PAGE of KcsA C-terminus cleavage, Figure S2: HSQC of KcsA proteoliposomes, Figure S3: CP-MAS NMR of KcsA proteoliposomes, Figure S4: Example of KcsA type assignment from hCCH-TOCSY data, Figure S5: Isopycnic gradient ultracentrifugation of KcsA proteoliposomes, Figure S6: Electron micrographs of KcsA liposomes, Figure S7: Lipid degradation in KcsA proteoliposome samples from TOCSY Data, Figure S8: Planes of intra-residue 3D HNCA of KcsA proteoliposomes, Figure S9: Planes of inter-residue (i + 1) 3D HNCO of KcsA proteoliposomes, Figure S10: Residue types identified in hCCH-TOCSY data of KcsA, Figure S11: HR-MAS sample radius, Table S1: KcsA ^13^C *T*_2_s, Table S2: ^1^H-^13^C HSQC per sample chemical shifts and relative intensities of various species, Table S3: KcsA proteoliposome sample details, and Detailed Materials and Methods [59,60,61,62,63,64,65].
