# Supplementary material for: High-Resolution Magic Angle Spinning NMR of KcsA in Liposomes: The Highly Mobile C-Terminus
Source: Biomolecules. 2022 Aug 15;12(8):1122. doi: 10.3390/biom12081122 (PMC9405666; doi:10.3390/biom12081122)
Supplement: Supplementary file 1 [file biomolecules-12-01122-s001.zip › biomolecules-1813427-supplementary.pdf]

High-Resolution Magic Angle Spinning NMR of KcsA in Liposomes: The Highly Mobile C-Terminus

Gary S. Howarth and Ann E. McDermott *

Supplementary Information

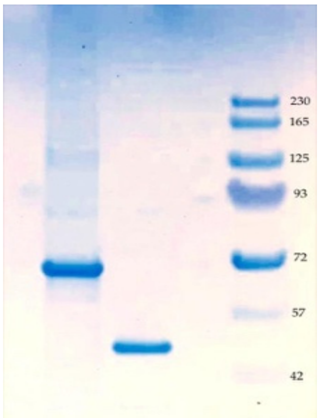

Figure S1: SDS PAGE of KcsA C-terminus cleavage
Full-length KcsA (left), KcsA-Δ125 from chymotrypsin reaction (center), and protein ladder (right). Full length tetramer approximately 70 kDa, expected cleavage product = 53.5 kDa.

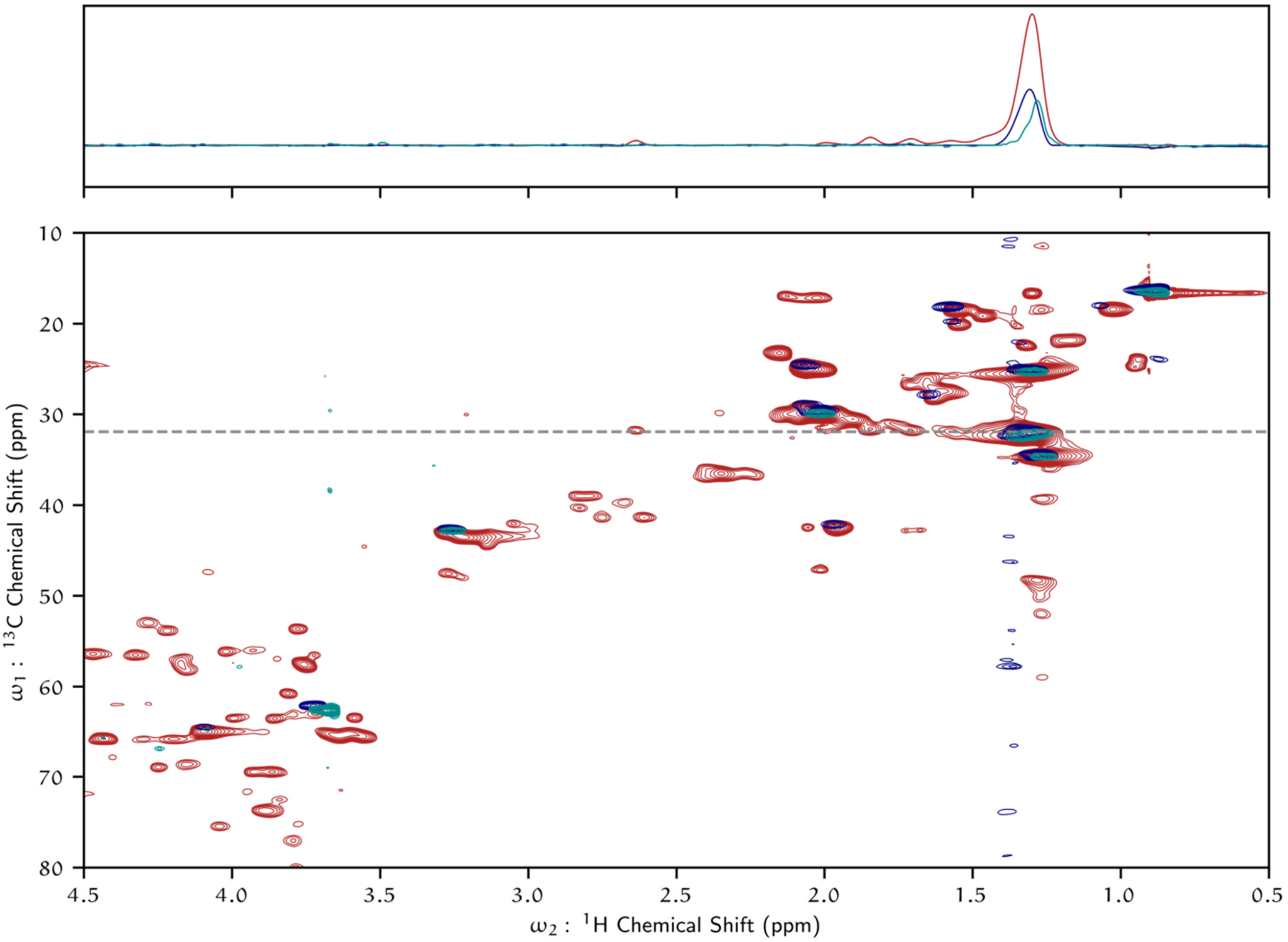

Figure S2: HSQC of KcsA proteoliposomes
Superimposed ^{1}H–^{13}C HSQC by HR-MAS of full-length KcsA in DOPE-DOPS liposomes (red), KcsA–Δ125 in DOPE- DOPS liposomes (navy), and DOPE-DOPS liposomes (cyan), showing that most resonances in full-length KcsA originate from residues 125-160. Slice of data at dash line (^{13}C: 42 ppm) displayed at top, showing PE- (^{1}H: 3.25 ppm) signal intensity is similar for all three samples. All samples: pH 7.25, 50 mM K^{+} 308 K, 5 kHz MAS.

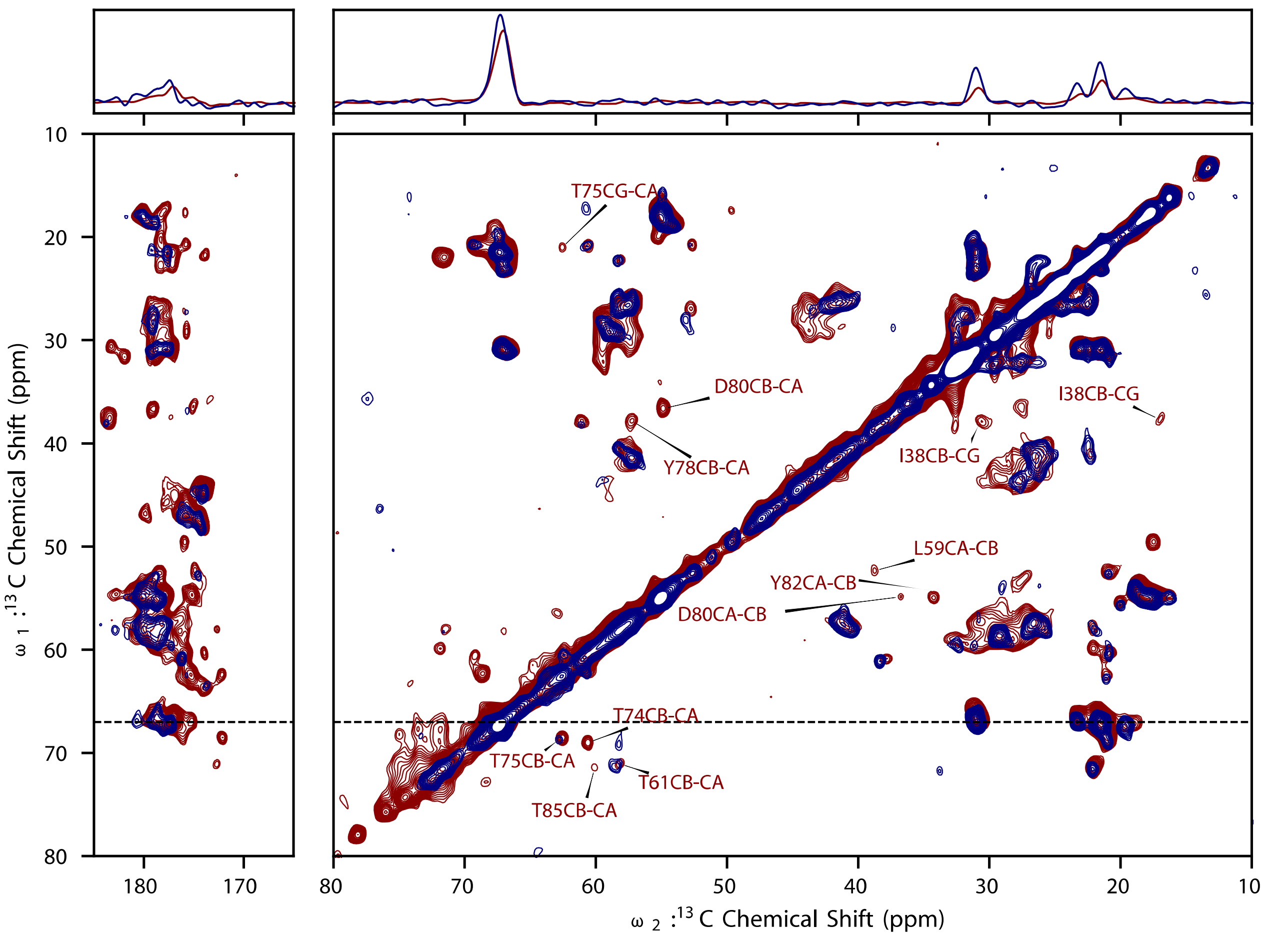

Figure S3: CP-MAS NMR of KcsA proteoliposomes
Superimposed ^{13}C–^{13}C proton-driven spin diffusion (DARR) CP-MAS full-length KcsA in DOPE-DOPS liposomes (red), KcsA-Δ125 in DOPE-DOPS liposomes (navy), showing the transmembrane domain of KcsA-Δ125 is folded and ^{13}C enriched. Assignments displayed are based on prior studies of full-length constructs at similar conditions. Slice of data at dashed line (^{13}C: 78 ppm) displayed on top. 50 ms DARR mixing period, pH 7.25, 50 mM K^+, 275 K, 16.6 kHz MAS.

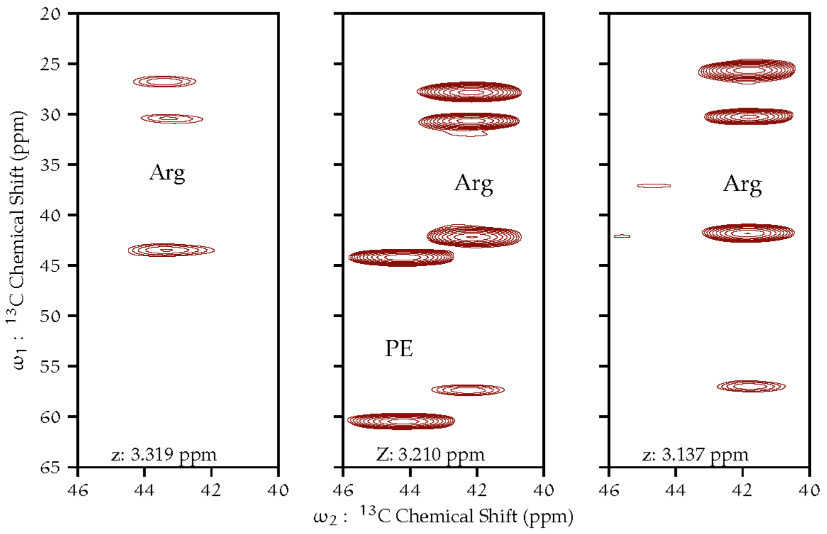

Figure S4: Example of KcsA type assignment from hCCH-TOCSY data

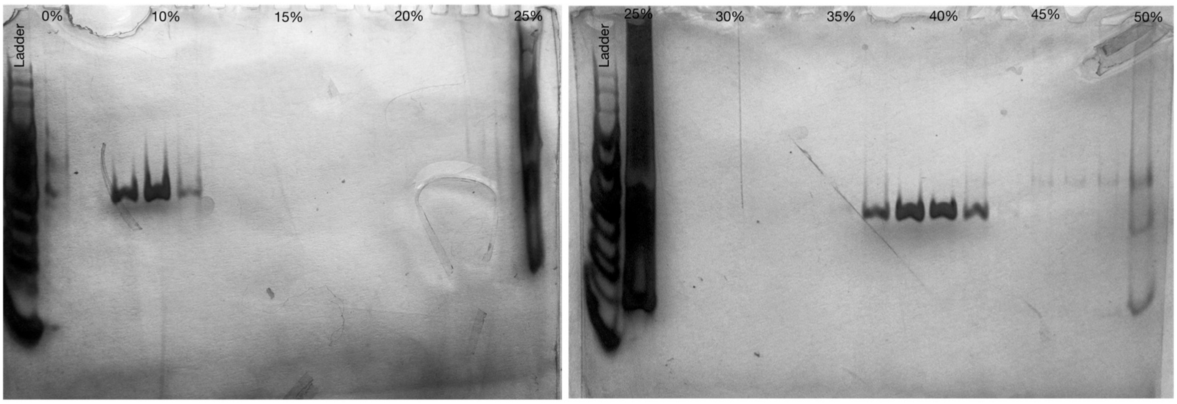

Figure S5: Isopycnic gradient ultracentrifugation of KcsA proteoliposomes
Silver-stained SDS-PAGE gels of aliquots, from two columns, KcsA ULVs on a sucrose gradient column subjected to ultracentrifugation at 107,000•*g* (max), with sucrose content indicated.

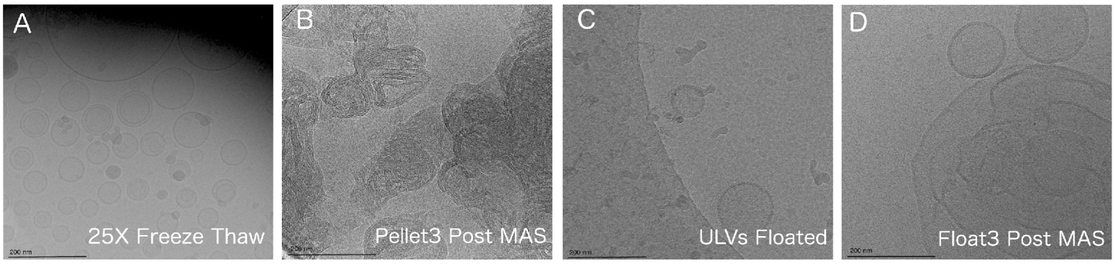

Figure S6: Electron micrographs of KcsA liposomes
Cryo-electron micrographs of KcsA liposomes. LPR = 1, 9:1 DOPE-DOPS. (A) After 25X freeze thaw (B) Sample 'A' after approximately 160 hours 5kHz MAS 308K, (C) Sample 'A' after addition of sucrose, (D) Sample 'C' after approximately 160 hours 5kHz MAS 308K

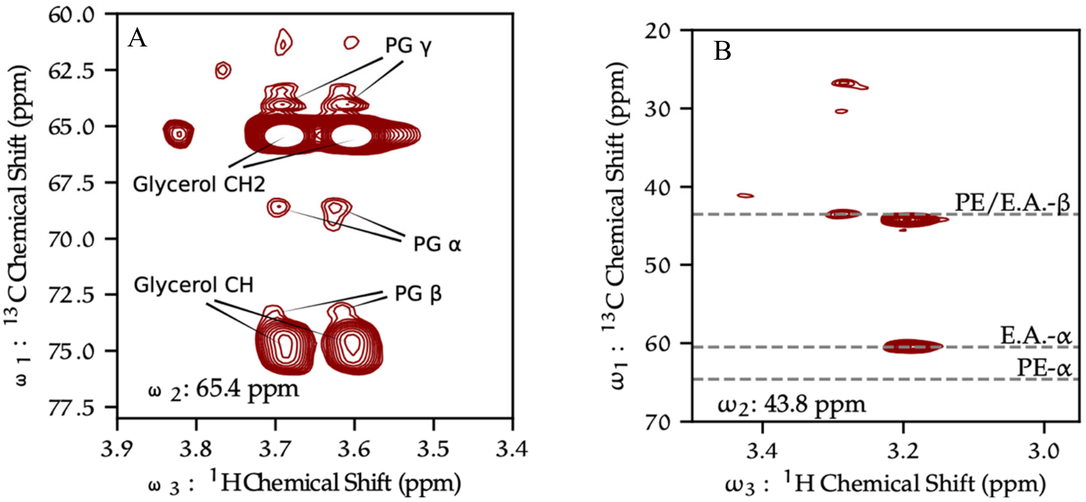

Figure S7: Lipid degradation in KcsA proteoliposome samples from TOCSY Data
(A) Slice of hCCH-TOCSY 3D correlation at 64.4 ppm. Minor resonances from PG and major resonances from free glycerol are labelled. (B) Slice of hCCH-TOCSY 3D correlation at 43.8 ppm. Expected ^{13}C shifts for phosphoethanol amine related species indicated.

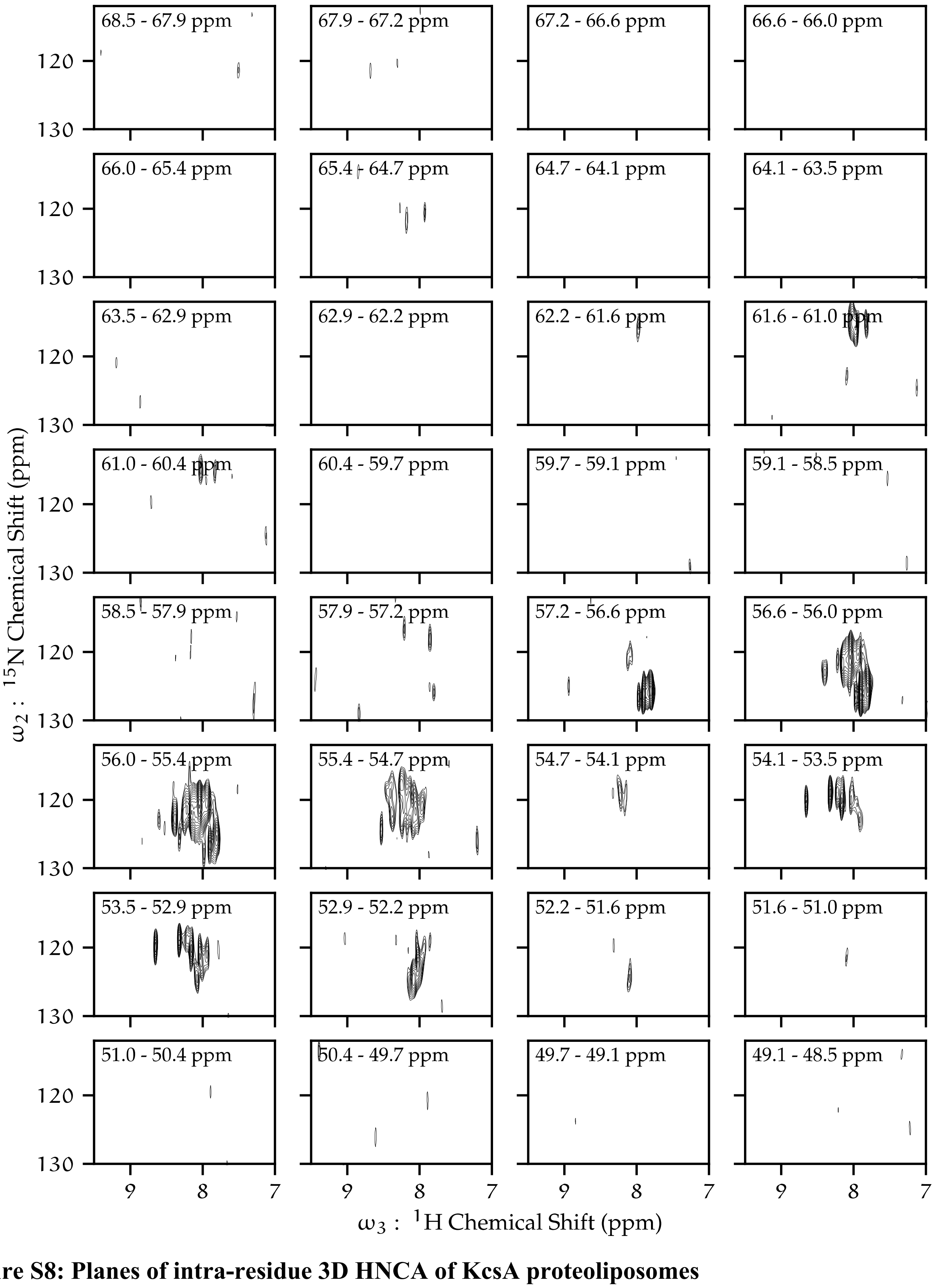

Figure S8: Planes of intra-residue 3D HNCA of KcsA proteoliposomes

U-13C,15N- KcsA pH 4.0, 50 mM K+. Z-axis (13C (ppm)) range is annotated on each plane. 9 kHz MAS, 308 K.

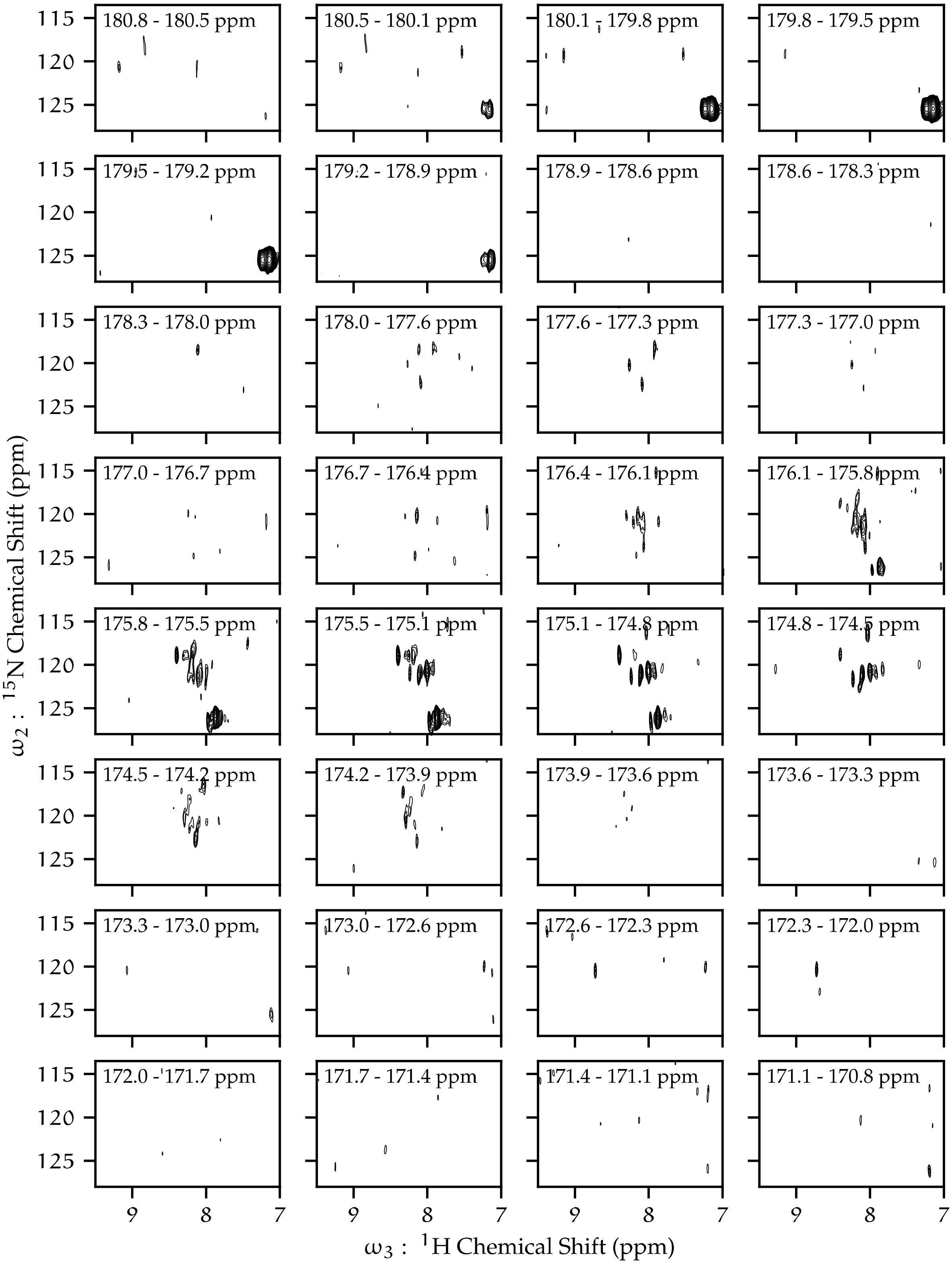

Figure S9: Planes of inter-residue (i+1) 3D HNCO of KcsA proteoliposomes
Fractionally deuterated U-13C,15N- KcsA at pH 4.0, 50 mM K+. Z-axis (13C (ppm)) range is annotated on each plane. 9 kHz MAS, 308 K

N-TERMINUS

1							2	3	4	5	6	7	8	9	10	11	12	13	14	15	16	17	18	19	20
M	H	H	H	H	H	H	P	P	M	L	S	G	L	L	A	R	L	V	K	L	L	L	G	R	H

TRANSMEMBRANE DOMAIN

21	22	23	24	25	26	27	28	29	30	31	32	33	34	35	36	37	38	39	40	41	42	43	44	45	46	47	48	49	50	51	52	53	54	55	56	57	58	59	60	61	62	63	64	65	66	67
G	S	A	L	H	W	R	A	A	G	A	A	T	V	L	L	V	I	V	L	L	A	G	S	Y	L	A	V	L	A	E	R	G	A	P	G	A	Q	L	I	T	Y	P	R	A	L	W

68	69	70	71	72	73	74	75	76	77	78	79	80	81	82	83	84	85	86	87	88	89	90	91	92	93	94	95	96	97	98	99	100	101	102	103	104	105	106	107	108	109	110	111	112	113	114	115
W	S	V	E	T	A	T	T	V	G	Y	G	D	L	Y	P	V	T	L	W	G	R	L	V	A	V	V	V	M	V	A	G	I	T	S	F	G	L	V	T	A	A	L	A	T	W	F	V

C-TERMINUS

116	117	118	119	120	121	122	123	124	125	126	127	128	129	130	131	132	133	134	135	136	137	138	139	140	141	142	143	144	145	146	147	148	149	150	151	152	153	154	155	156	157	158	159	160
G	R	E	Q	E	R	R	G	H	F	V	R	H	S	E	K	A	A	E	E	A	Y	T	R	T	T	R	A	L	H	E	R	F	D	R	L	E	R	M	L	D	D	N	R	R

Residues identified in hCCH-TOCSY data

E	F/Y	K	L	R
2	2	1	2	3

Figure S10: Residue types identified in hCCH-TOCSY data of KcsA (Table 1) mapped on to KcsA sequence

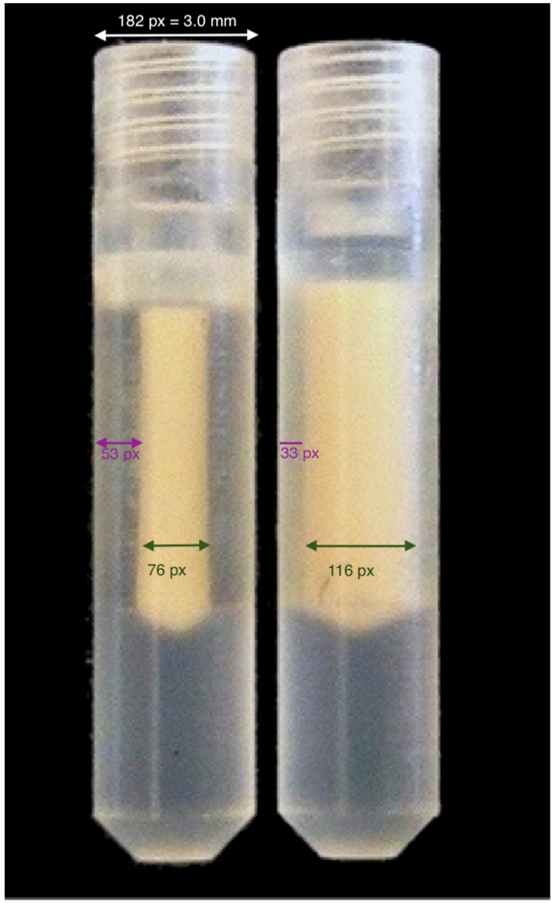

Figure S11: HR-MAS Sample Radius

Single image of two rotor inserts containing post-experiment KcsA proteoliposomes. Left: ‘floating’ sample in sucrose-augmented buffer, and Right: ‘sinking’ sample with no sucrose in buffer. Rotor insert width is 3.0 mm. We measured the sample radius using pixels from this image, obtaining a sample radius of 0.63 mm for the floating sample and 0.96 mm for the pelleted sample.

Table S1: KcsA ^{13}C T_2s

$^{13}C\alpha$ T_2 U-^{1}H,^{13}C,^{15}N-KcsA in 9:1 DOPE-DOPS liposomes, pH 4.0, 50 mM K^+, 308K, 5 kHz MAS. Lipids assigned from chemical shifts, other resonances assumed to be protein H-Cα resonances. In the absence of relaxation, the optimal transfer time is $1/(2J)$. $J_{N\text{-}C\alpha}$ is typically 7-11 Hz.

Site	Type	^{1}H (ppm)	^{13}C (ppm)	^{13}C T_2 (ms)
	protein	4.04	51.4	3.6
	protein	3.98	54.6	3.4
Arg-α	protein	4.03	55.2	2.8
Met-α	protein	4.30	55.8	3.9
	protein	4.16	56.9	2.7
Lys-α	protein	3.72	56.9	2.8
	protein	4.37	61.6	2.2
Thrβ	protein	4.19	69.0	2.4
PE-β	lipid	3.24	43.9	6.5
PE-α	lipid	4.05	64.2	5.9
PG-γ	lipid	3.56	64.7	3.7
PG-γ	lipid	3.63	64.7	3.8
PG-α	lipid	3.82	68.9	4.3
PG-α	lipid	3.88	68.9	4.4
PG-β	lipid	3.80	73.1	7.2

Table S2: ^{1}H-^{13}C HSQC chemical shifts n various samples and relative intensities of various species

^{1}H-^{13}C HSQC chemical shifts of exogenous lipid signals in KcsA liposome samples by HR-MAS. "–" indicates no detected signal for given peak and "o" indicates the presence of grossly overlapping peaks in peak region. Glycerol CH_2 is degenerate with PG γ signal. All samples reconstituted in natural abundance phospholipids with dioleoyl fatty acids chains with lipid-to-protein ratio of 1 (w/w), 308 K, 5 kHz MAS, internally referenced to DSS. Sample details given in Table S3.

ID	Protein	Lipid	pH	PG α<br>^{1}H (ppm) / ^{13}C (ppm)	PG β	PG γ & Glycerol CH_2†	Glycerol CH	HC=C
A	(empty)	PE/PS	7.3	–	–	–	–	–
B	KcsA	PE/PS	7.3	3.94/73.7 ; 3.88/68.4	3.94/68.5	3.69/65.4 ; 3.61/65.4	3.83/74.9	5.1/126.9
D	KcsA	PE/PS	6.3	3.92/69.4 ; 3.86/69.4	3.89/73.7	3.63/65.4 ; 3.61/65.3	–	5.09/127.1
E	KcsA	PE/PS	4	3.92/69.6 ; 3.85 / 69.6	3.89/73.8	3.66/65.2 ; 3.6/65.2	–	–
F	^{2}H KcsA	PE/PS	6.3	–	–	3.64/65.5 ; 3.55/65.5	–	–
G	^{2}H KcsA	PE/PS	4	–	3.95/73.6	3.71/65.4	3.8/74.5	–
H	KcsA–ΔC	PE/PS	7.3	–	–	–	–	–
I	KcsA	PE/PS	7.3	3.94/69.1 ; 3.89/69.1	3.93/73.5	3.67/65.4 ; 3.58/65.4	o	–
J	KcsA	PE/PS	7.3	3.95/69.4 ; 3.88/69.3	3.92/73.6	o	o	–
K	KcsA	PE/PS	7.3	o	o	3.66/65.4 ; 3.57/65.4	o	–
L	KcsA	PE/PS	6.3	3.98/69.3 ; 3.91/69.3	3.96/73.6	3.7/65.5 ; 3.61/65.4	o	5.11/126.9
M	KcsA	PC	4	3.92/73.8	3.96/69.5	3.7/65.3 ; 3.63/65.3	–	5.05/127

Table S3: KcsA proteoliposome sample details

Description of various samples used in HR-MAS samples, providing details for **Table S2:** H-^{13}C HSQC chemical shifts

ID	Protein	Labeling	Lipids	pH	sucrose (% w/w)	buffer	K^+ (mM)	Mg^{2+} (mM)	D_2O (% vol.)
A	no protein		DOPE/DOPS	7.3	0	tris	50	0	15
B	KcsA	U-^{13}C^{15}N	DOPE/DOPS	7.3	0	tris	50	0	~90
D	KcsA	U-^{13}C^{15}N	DOPE/DOPS	6.3	0	pipes	50	0	10
E	KcsA	U-C^{13}C^{15}N	DOPE/DOPS	4	0	citrate	50	0	10
F	KcsA	F–^{2}H U-^{13}C^{15}N	DOPE/DOPS	6.3	0	pipes	50	0	10
G	KcsA	F–^{2}H U-^{13}C^{15}N	DOPE/DOPS	4	0	citrate	50	0	7
H	KcsA–ΔC	U-^{13}C^{15}N	DOPE/DOPS	7.3	0	tris	50	0	~90
I	KcsA	U-^{13}C^{15}N	DOPE/DOPS	7.3	38	tris	50	2	10
J	KcsA	U-^{13}C^{15}N	DOPE/DOPS	7.3	42	tris	50	0	10
K	KcsA	U-^{13}C^{15}N	DOPE/DOPS	7.3	44	tris	50	2	10
L	KcsA	U-^{13}C^{15}N	DOPE/DOPS	6.3	45	tris	50	0	10
M	KcsA	U-^{13}C^{15}N	DOPC	4	0	citrate	150	0	~20

Detailed Materials and Methods

Protein Expression and Purification

Wild-type KcsA was expressed as an N-terminal His_6 fusion protein cloned onto a PASK90 plasmid, which confers ampicillin resistance, and was over-expressed in *Escherichia coli* cells using established protocols developed by our group [1]. The plasmid was transformed into chemically competent JM83 cells by heat shock at 42 °C and plated onto Luria broth (LB) (Sigma-Aldrich, Saint Louis, MO, USA), agar plates augmented with 100 mg L^{-1} carbenicillin overnight at 37 °C. Individual colonies were selected and used to inoculate several precultures of 5 mL of carbenicillin LB media and grown to an $OD_{600} \approx 1$. Four 1L LB cultures were inoculated using one preculture each and allowed to grow at 37 °C and 250 RPM of shaking to $OD_{600} \approx 0.9$, at which point the cultures were harvested by centrifugation at 4×10^3 RCF for 10 min. The cells were then resuspended in 1L M9 minimal media whose sole source of carbon was U-^{13}C glucose and sole nitrogen source was ^{15}N ammonium chloride; see [1] for the complete recipe. The JM83 cell line is a proline auxotrophic, so natural-abundance proline was added to the M9. The cells were incubated for 60 min at 37 °C and 250 RPM of shaking in M9 media. The culture temperature was lowered to 25 °C and the shaking rate increased to 300 RPM. Protein expression was then initiated by the addition of 1 μg L^{-1} anhydrotetracyclin (aTC) (Sigma-Aldrich, Saint Louis, MO, USA), a synthetic lactose mimic, and the culture was allowed to express overnight. Cells were then harvested by centrifugation at 4×10^3 RCF for 30 min and frozen at −80 °C until further processing.

Expression of fractional deuteration with uniform ^{13}C and ^{15}N KcsA was accomplished with minor modifications from [2], which in turn was an adaptation from [3]. Briefly, KcsA culture was prepared as described above using Bl21(DE3) *E. coli* cells (New England Biolabs, Ipswich, MA, USA). Cells were grown at 35 °C, with 250 RPM shaking, in 1L of LB selection medium (using carbenicillin) to an $OD_{600} = 0.8$ and were collected by centrifugation. The cell pellet was rinsed in approximately 50 mL of D_2O (Cambridge Isotopes, Cambridge, MA, USA). The cells were then suspended in 500 mL of M9 medium solution described with the exception that instead of using deionized water to prepare the M9 minimal media, 98% D_2O was used. The medium, as shown below, was supplemented with 3g U-^{13}C-glucose and 1 g ^{15}N-ammonia. This culture was allowed to grow for 1 h; then, the temperature was lowered to 25 °C and shaking increased to 300 RPM and was induced with aTC. The cells were allowed to express for 10 h and harvested by centrifuge and stored at −80 °C.

KcsA was purified in the same manner regardless of labelling strategy. Frozen cells were resuspended in buffer 50 mM tris base, 150 mM potassium chloride, and 2 mM decyl-β-maltopyranoside (Anatrace, Maumee, OH, USA) detergent (DM), pH 7.5, using approximately 5mL buffer per gram of cell pellet. The cells were then lysed by passage through a French Pressure cell operating at 10,000–20,000 PSI three to five times until lysate became dark and highly viscous. Protease inhibitor cocktail (Pierce) and 10% DM (*w*/*w*) per cell pellet mass were added to the lysate, which was incubated overnight at 4 °C while rocking. The lysate was then centrifuged at 2.5×10^5 RPM and the resulting pellet, lipids and other cell debris were discarded. The crude lysate was sterile-filtered and purified by His-Select nickel affinity (Sigma-Aldrich, Saint Louis, MO, USA) chromatography using buffer consisting of 50 mM tris base, 300 mM potassium chloride, and 5 mM DM, pH 7.5 as equilibrium buffer, with 40 mM imidizole in the wash buffer and 300

mM in the elution buffer. The presence and purity of the resulting KcsA was determined by SDS-PAGE with a characteristic band for the properly folded tetramer appearing at 70 kDa. Imidazole was removed by a Amicon spin concentrator (MilliporeSigma, Burlington, MA, USA) or Sephadex G-25 desalting column (Sigma-Aldrich, Saint Louis, MO, USA). The concentration of the protein was determined by UV-Vis spectroscopy at 280 nm with a calculated extinction coefficient of 33,570 M^{-1} cm^{-1} [4]. The purified, concentrated KcsA was stored at 4 °C and reconstituted with lipids within a matter of hours.

KcsA Reconstitution

Liposomes were formed from a 9:1 mass ratio of 1,2-dioleoyl-sn-glycero-3-phosphoethanolamine (DOPE) to 1,2-dioleoyl-sn-glycero-3-phospho-L-serine (DOPS), which were obtained as a chloroform solution (Avanti, Alabaster, AL, USA), dried as a thin film under N_2 ga, resolubilized in n-hexane (Sigma-Aldrich, Saint Louis, MO, USA), dried again under N_2 gas and solubilized by bath sonication in 10 mM DM, 50 mM Tris, and 100 mM KCl, pH 7.5. Lipids were mixed with KcsA in a 1:1 ratio by mass, diluted to 2 mM DM and dialyzed in 30 kDa MWCO tubing (Spectrum Chemical, New Brunswick, NJ, USA) with three exchanges of 4 L of buffer at 12–18 h intervals at room temperature. Proteoliposomes were harvested by centrifugation at 5700 RCF for 30 min and then stored at −80 °C. The presence of KcsA as a tetramer in the liposomes was verified by SDS-PAGE before and after NMR experiments.

NMR

J-coupled-based experiments were performed on a Bruker magnet with a proton field of 750 MHz using a 4 mm high-resolution magic angle spinning probe (HR-MAS) equipped with $^{1}H/^{13}C/^{15}N/^{2}H$ channels and a 40 G/cm gradient coil oriented along the magic angle. Experiments were generally performed between 4–5 kHz MAS and 308 K unless stated otherwise. Typical hard pulses were 31 kHz for ^{1}H, 33 kHz for ^{13}C, and 20 kHz for ^{15}N. Decoupling fields and spinlocks were typically 10 kHz. Heteronuclear decoupling was accomplished using WALTZ16 [6]. HSQCs were phase-sensitive using double inept transfer with preservation of equivalent pathway (PEP) sensitization, trim pulses (100 μs), echo/antiecho-TPPI gradient selection, and homonuclear decoupling during acquisition using the Bruker sequence hsqcetgpsi for ^{13}C-resolved spectra, and hsqcetf3gpsi2 for ^{15}N resolved spectra [7–9]. hCCH-TOCSY spectra were collected with full-rotor period synchronized TOCSY spinlocks using the Bruker sequence hcchdigp3d2. Site-specific T_2 measurements were collected by adding a single rotor-synchronized ^{13}C spin-echo between the two inept transfers and increasing the delay of the echo over at least five steps until magnetization had decayed by at least 90%.

Quantitative ^{1}H spectra were collected with calibrated ^{1}H 90° (typically ~9 μs), with recycle delays of at least 5 s (the longest T_1 in samples was typically ~0.9 s) with pre-saturation on the H_2O resonance during the recycle delay and a presaturation field strength of approximately 25 Hz. Typically, 4 to 16 scans were collected to reach a signal-to-noise ratio of more than 20 for the CH_2 region.

Cross-polarization MAS was performed with a Bruker 750 Avance I spectrometer using a 3.2 mm $^{1}H/^{13}C/^{15}N$ e-free probe at 16.6 kHz at 275 K rotor set temperature. Typical field strengths were 100 kHz for ^{1}H and 50 kHz for ^{13}C. Acquisition times in the direct dimension were approximately 20 ms collected in 2048 points and in the indirect dimension were typically 4 ms in 128 points. All ^{13}C-acquired data were zero-filled to twice the number of points acquired and were multiplied by Lorentzian-to-Gaussian function with 10–40 Hz of line broadening and 0.3–0.01 Gaussian factors applied empirically in the direct dimension, and the indirect dimension data were multiplied by SIN^2 function of pure cosine phase.

Solution NMR was performed on a Bruker 500 Ascend instrument using a $^{1}H/^{13}C/^{15}N$ probe. Sample temperatures were 300 K. Typical field strengths were 18 kHz for ^{1}H and 8 kHz for ^{13}C for hard pulses, and 2 kHz for ^{13}C heteronuclear decoupling using WALTZ16. Spoil gradients were accomplished with 7.6 T m^{-1} of 1 ms duration. HSQCs were phase-sensitive and multiplicity-edited using double INEPT transfer, trim pulses (1 ms), PEP, and used shaped pulses for inversion on ^{13}C (500 µs) with echo/antiecho-TPPI gradient selection and decoupling during acquisition (Bruker sequence: hsqcedetgpsp.3). Direct dimensions were acquired for 50 ms in 2048 points and indirect dimensions were acquired for approximately 10 ms in 512 points. The 3D TOCSY data were always acquired with ^{1}H as the direct dimension for 100 ms in 2048 points, while indirect dimensions of ^{1}H were acquired for 7.5 ms in 128 points, and ^{13}C for 2.5 ms in 64 points, with states-TPPI detection mode. HcCH-TOCSY indicates ^{13}C as F2 and ^{1}H as F1 and hCCH-TOCSY indicates ^{13}C in both indirect dimensions. All proton-acquired data were zero-filled to twice the number of points. FIDs were multiplied by SIN^2 function of pure cosine phase.

KcsA Cleavage Preparation

To cleave the C-terminus, 1 mg mL^{-1} KcsA in 5 mM DM was incubated with 20 µg mL^{-1} of bovine α-chymotrypsin (Sigma-Aldrich, Saint Louis, MO, USA) for 3 h at 35 °C [10]. KcsA was isolated using Ni-affinity gel as described above. An aliquot of the full-length construct and the post-reaction purified KcsA were analyzed by SDS-PAGE using a 4–12% Bis-Tris mini gel (Thermo Fisher) at 200 V for 35 min. BLUeye protein ladder (Sigma-Aldrich, Saint Louis, MO, USA) was used as a standard. The gel was then stained with PageBlue (Thermo Scientific, Waltham, MA, USA) Coomassie brilliant blue stain according to manufacturer direction. Individual bands were then cut from the gel and placed into new centrifuge tubes and delivered within two hours, on ice, and sent to the proteomics core for mass spectrometry, confirming expected cleavage products.

Sucrose Gradients

Gradients were prepared by layering equal volumes of two sucrose concentrations (depending on upper and lower bounds of gradient desired) in buffer of 50 mM Tris and 50 mM KCl, pH 7.25 in 12 mL ultracentrifuge tubes (Beckman), then applying a preprogramed algorithm to mix the layers using a Gradient Master (BioComp Instruments, Fredericton, NB, CA) and cooling to 4 °C overnight. The linearity of the gradient formation protocol and of aliquots described below was verified by measuring the density of tube fractions using a calibrated pipette with low retention tips and analytical balance. For highly viscous samples, or when the sample refused to entirely leave the tip, mass was determined by difference with the solution in the pipette tip. Proteoliposomes were prepared as described above except, a rhodamine-conjugated lipid (1,2-dioleoyl-sn-glycero-3-phosphoethanolamine-N-(lissamine rhodamine B sulfonyl) (Rhod-PE) (Avanti, Alabaster, AL, USA) was added to the lipid mixture to more easily visualize and quantify the mixtures. The lipid to protein ratio was 1:1 by mass. The lipid mixture was 900:10:1 PE-PS-Rhod PE by mass. A total of 20 mg of KcsA proteoliposomes was suspended in 0.5 mL 5% sucrose solution then added to the top of the gradient. The tube was subjected to ultracentrifugation, with proper counterweighting, in a Beckman Coulter L70 centrifuge using a swinging bucket rotor (SW41) at 25,000 RPM, corresponding to a relative centrifugal field ranging from 47,200× *g* to 107,000× *g*, for 24 h at 4 °C with the slowest acceleration and no brake during deceleration. After centrifugation, a cloudy band of faint pink is visible in the lower third of the tube, indicating the presence of the proteoliposomes at that layer. The bottom of the tube was pierced with a 20 Ga needle that was then

removed and the tube was allowed to flow under gravity at 4 °C. Fractions of 0.5 mL were collected until the proteoliposome band approached the bottom of the tube when individual drops (~0.1 mL) were collected. Fractions were measured by UV-VIS at 280 nm for the presence of protein and 560 nm for the presence of lipids as a qualitative measure. Background scattering caused by the lipids renders 280 nm absorbance sufficient to detect the presence of protein but not to quantify it. To quantify protein, we adapted a procedure using bromophenol blue and Triton-X100 from Greenberg and Craddock [11]. Specifically, we formed the assay reagent by mixing 25 mg of bromophenol blue (Sigma-Aldrich, Saint Louis, MO, USA), 20 mL of ethanol (HPLC grade, Sigma-Aldrich, Saint Louis, MO, USA), 3.0 mL of glacial acetic acid (Fischer, Waltham, MA, USA), 5 mL Triton-X100 (Sigma-Aldrich, Saint Louis, MO, USA), and 250 mL of deionized water. To implement the assay, we mixed the reagent with the analyte in a 9:1 volume ratio, bath sonicated at 35 °C for 5 min and measured the UV-VIS absorbance at 610 nm and background subtracted absorbance of the regent and deionized water. We used this reagent to develop a standard curve from a stock of KcsA at 1.0 mg/mL in 10 mM DM with 15 concentrations of KcsA solution in triplicate ranging from 0.10 µg mL^{-1} to 20 µg mL^{-1}, finding a linear response of the assay to KcsA in this range. We investigated the ability of sucrose to interfere with assay and found no significant difference at sucrose concentrations less than 50% (*w*/*w*). SDS-PAGE, as described above, with a Pierce silver stain kit (ThermoFisher, Waltham, MA, USA) was used to visualize protein in gradient fractions. Total lipids were extracted using the procedure described in [12]. Lipids were quantified using a standard curve based on UV-VIS absorbance of Rhod-PE at 560 nm.

Supplemental Information References

[1] Bhate, M.P.; Wylie, B.J.; Thompson, A.; Tian, L.; Nimigean, C.; McDermott, A.E. Preparation of Uniformly Isotope Labeled KcsA for Solid State NMR: Expression, Purification, Reconstitution into Liposomes and Functional Assay. *Protein Expression and Purification* **2013**, doi:10.1016/j.pep.2013.07.013.

[2] Mance, D.; Sinnige, T.; Kaplan, M.; Narasimhan, S.; Daniëls, M.; Houben, K.; Baldus, M.; Weingarth, M. An Efficient Labelling Approach to Harness Backbone and Side-Chain Protons in 1H-Detected Solid-State NMR Spectroscopy. *Angew. Chem.* **2015**, *127*, 16025–16029. https://doi.org/10.1002/ange.201509170.

[3] Shekhtman, A.; Ghose, R.; Goger, M.; Cowburn, D. NMR Structure Determination and Investigation Using a Reduced Proton (REDPRO) Labeling Strategy for Proteins. *FEBS Lett.* **2002**, *524*, 177–182. https://doi.org/10.1016/S0014-5793(02)03051-X.

[4] Gill, S.C.; von Hippel, P.H. Calculation of Protein Extinction Coefficients from Amino Acid Sequence Data. *Anal. Biochem.* **1989**, *182*, 319–326.

[5] Heginbotham, L.; Kolmakova-Partensky, L.; Miller, C. Functional Reconstitution of a Prokaryotic K + Channel. *J. Gen. Physiol.* **1998**, *111*, 741–749. https://doi.org/10.1085/jgp.111.6.741.

[6] Shaka, A.J.; Keeler, J.; Freeman, R. Evaluation of a New Broadband Decoupling Sequence: WALTZ-16. *J. Magn. Reson.* **1983**, *53*, 313–340. https://doi.org/10.1016/0022-2364(83)90035-5.

[7] Schleucher, J.; Schwendinger, M.; Sattler, M.; Schmidt, P.; Schedletzky, O.; Glaser, S.J.; Sørensen, O.W.; Griesinger, C. A General Enhancement Scheme in Heteronuclear Multidimensional NMR Employing Pulsed Field Gradients. *J. Biomol. NMR* **1994**, *4*, 301–306. https://doi.org/10.1007/BF00175254.
[8] Palmer, A.G.; Cavanagh, J.; Wright, P.E.; Rance, M. Sensitivity Improvement in Proton-Detected Two-Dimensional Heteronuclear Correlation NMR Spectroscopy. *J. Magn. Reson.* **1991**, *93*, 151–170. https://doi.org/10.1016/0022-2364(91)90036-S.
[9] Kay, L.E.; Keifer, P.; Saarinen, T. Pure Absorption Gradient Enhanced Heteronuclear Single Quantum Correlation Spectroscopy with Improved Sensitivity. *J. Am. Chem. Soc.* **1992**, *114*, 10663–10665.
[10] Doyle, D.A.; Morais Cabral, J.; Pfuetzner, R.A.; Kuo, A.; Gulbis, J.M.; Cohen, S.L.; Chait, B.T.; MacKinnon, R. The structure of the potassium channel: molecular basis of K+ conduction and selectivity. *Science* **1998**, *280*, 69–77, doi:10.1126/science.280.5360.69.
[11] Greenberg, C.S.; Craddock, P.R. Rapid Single-Step Membrane Protein Assay. *Clin. Chem.* **1982**, *28*, 1725. https://doi.org/10.1093/clinchem/28.7.1725.
[12] Bligh, E.G.; Dyer, W.J. A Rapid Method of Total Lipid Extraction and Purification. *Can. J. Biochem. Physiol.* **1959**, *37*, 911–917. https://doi.org/10.1139/o59-099.
